# Bacterial Diversity in Meconium of Preterm Neonates and Evolution of Their Fecal Microbiota during the First Month of Life

**DOI:** 10.1371/journal.pone.0066986

**Published:** 2013-06-28

**Authors:** Laura Moles, Marta Gómez, Hans Heilig, Gerardo Bustos, Susana Fuentes, Willem de Vos, Leónides Fernández, Juan M. Rodríguez, Esther Jiménez

**Affiliations:** 1 Departamento de Nutrición, Bromatología y Tecnología de los Alimentos, Universidad Complutense de Madrid, Madrid, Spain; 2 Laboratory of Microbiology, Wageningen University, Wageningen, The Netherlands; 3 Servicio de Neonatología, Hospital Universitario 12 de Octubre, Madrid, Spain; 4 Department of Bacteriology and Immunology, University of Helsinki, Helsinki, Finland; Institute of Agrochemistry and Food Technology, Spain

## Abstract

The establishment and succession of bacterial communities in infants may have a profound impact in their health, but information about the composition of meconium microbiota and its evolution in hospitalized preterm infants is scarce. In this context, the objective of this work was to characterize the microbiota of meconium and fecal samples obtained during the first 3 weeks of life from 14 donors using culture and molecular techniques, including DGGE and the Human Intestinal Tract Chip (HITChip) analysis of 16S rRNA amplicons. Culture techniques offer a quantification of cultivable bacteria and allow further study of the isolate, while molecular techniques provide deeper information on bacterial diversity. Culture and HITChip results were very similar but the former showed lower sensitivity. Inter-individual differences were detected in the microbiota profiles although the meconium microbiota was peculiar and distinct from that of fecal samples. *Bacilli* and other *Firmicutes* were the main bacteria groups detected in meconium while *Proteobacteria* dominated in the fecal samples. Culture technique showed that *Staphylococcus* predominated in meconium and that *Enterococcus,* together with Gram-negative bacteria such as *Escherichia coli*, *Escherichia fergusonii, Klebsiella pneumoniae* and *Serratia marcescens*, was more abundant in fecal samples. In addition, HITChip results showed the prevalence of bacteria related to *Lactobacillus plantarum* and *Streptococcus mitis* in meconium samples whereas those related to *Enterococcus*, *Escherichia coli*, *Klebsiella pneumoniae* and *Yersinia* predominated in the 3^rd^ week feces. This study highlights that spontaneously-released meconium of preterm neonates contains a specific microbiota that differs from that of feces obtained after the first week of life. Our findings indicate that the presence of *Serratia* was strongly associated with a higher degree of immaturity and other hospital-related parameters, including antibiotherapy and mechanical ventilation.

## Introduction

The microbial colonization of the infant gastrointestinal tract is an essential process in the human lifecycle since interactions established between the microbiota and the host have important consequences for human health and disease [1]. Therefore, acquisition and diversity of the gut microbiota in term neonates have been the subject of several studies [2], [3], [4], [5], [6], [7]. Different factors, such as mode of delivery, antibiotherapy, diet or environment, affect infant gut colonization [8], [9] although their actual contribution to shape the infant microbiota remains unclear. In addition, gestational age and weight at birth also exert a strong influence on this process. Previous studies monitoring the bacterial communities in preterm infants indicated that the fecal microbiota of premature infants is different compared with that of term infants [10], [11], [12], [13], [14], [15], [16], [17]. In fact, the gut colonization pattern of preterm infants has been described as delayed and aberrant [18]. Abnormal intestinal colonization during the first weeks of life may alter the barrier, nutritional and immunological functions of the host microbiota [19], [20] and, as a consequence, increases susceptibility to disease [21], [22]. Recently, a study on the bacterial diversity of meconium in six preterm infants showed an association between low bacterial diversity in meconium and high risk to develop sepsis [23]. In general, studies on the gut microbiota of preterm and term infants have been focused on feces; in contrast, information on the change of bacterial composition from meconium to feces during the first weeks of life is scarce, particularly in relation to preterm babies [12], [14]. Traditionally, it has been considered that the intestinal tract was sterile at birth, being rapidly colonized with microorganisms from the mother and the surrounding environment. However, some studies suggest that, actually, the meconium from healthy hosts is not sterile and that gut colonization may start before birth [4], [14], [24], [25], [26], [27], [28]. Therefore, studies on the bacterial diversity of meconium may provide new clues on the initial gut colonizers and their potential role in infant health and disease. In a previous work of our group [27] the microbial composition of meconium samples from term healthy babies born at the Hospital Universitario 12 de Octubre was studied by culture methods. Identification of isolates from different growth media revealed that enterococci were the predominant genera followed by staphylococci, *Escherichia coli* and *Enterobacter* spp. This microbiota was substituted by obligate anaerobes, such as bifidobacteria, that became predominant during the first week of life (unpublished data). In this context, the objective of this work was to analyze bacterial diversity in meconium and feces of preterm infants during their first month of life. For this purpose culture-dependent and culture-independent methods were used since they often provide complementary views on the microbial diversity of biological samples.

## Materials and Methods

### Patients and Sampling

The prospective study included 14 preterm babies born at the Hospital Universitario 12 de Octubre, Madrid (Spain) (Table 1). Written informed parental consent was obtained for each preterm before inclusion. To be eligible for enrolment, preterms must have been born at a gestational age ≤ 32 weeks and/or with birth weight ≤ 1,200 g, and without malformations or metabolic diseases. Relevant clinical data recorded for each infant, such as length of antibiotherapy, parenteral nutrition, nasogastric feeding, mechanical ventilation, hospital stay and type of feeding, are described in Table 2. All infants were fed with human milk (donor milk and/or their own mother’s milk) and, occasionally, with preterm formula.

**Table 1:** Demographic data for the infant cohort.

Infant	Gestational age (week)	Delivery mode	Gender	Birth weight (g)	Sample collection(day of life)
1	31	Vaginal	Male	2,190	0, 7, 14, 21
2	30	Cesarean section	Male	1,550	0, 7, 14, 21
3	27	Cesarean section	Female	1,080	0, 7, 14, 21
4	30	Cesarean section	Male	2,030	0, 7, 14, 21
5	30	Vaginal	Male	1,760	0, 7, 14, 21
6	24	Cesarean section	Female	600	0, 14, 21
7	27	Vaginal	Male	1,540	0, 7, 14, 21
8	26	Cesarean section	Female	790	0, 7, 14, 21
9	32	Vaginal	Female	1,310	0, 7, 14, 21
10	26	Vaginal	Female	920	0, 7, 14, 21
11	29	Cesarean section	Female	1,040	0, 7, 14, 21
12	31	Vaginal	Female	1,430	0, 7, 14, 21
13	24	Cesarean section	Female	750	0, 7, 21
14	27	Vaginal	Female	1,040	0, 14, 21

**Table 2:** Clinical characteristics of the preterm infants recruited in this study.

Infant	Hospital stay (days)	Antibiotherapy (days)	Mechanical ventilation (days)	Parenteral nutrition (days)	Nasogastric feeding tube (days)
1	26	3	0	3	18
2	42	3	0	5	38
3	60	4	0.5	3	48
4	27	3	2	0	26
5	27	0	0	0	26
6	113	5	9	8	107
7	68	4	2	5	58
8	84	7	0.5	6	70
9	28	3	0	0	21
10	102	15	26	8	97
11	47	2	0	5	45
12	37	3	0	4	35
13	92	8	1	14	83
14	68	7	0	7	62
Mean (95% CI)	58.64 (41.35; 75.93)	3.5 (3.0–7.0)*	0.25 (0.00–1.75)*	4.85 (2.66; 7.05)	52.40 (36.03; 68.82)

* Median (IQR).

First spontaneously evacuated meconium and fecal samples were collected by the medical staff of the Department of Neonatology of the hospital. Fecal samples were collected weekly from the diapers of the infants during their stay at the neonatal intensive care unit (NICU). All the samples were stored at −20°C until analysis. A non-used diaper was placed inside one of the incubators to be used as a control.

### Ethics Statement

The Ethical Committee on Clinical Research of the Hospital Clínico San Carlos of Madrid approved all study protocols (10/017-E). Samples and clinical information were obtained after informed, written consent by the study infants’ legal guardians.

### Culture Analysis of the Samples

Adequate dilutions of meconium and stool samples were spread onto Man, Rogosa and Sharpe (MRS; Oxoid, Basingstoke, UK) and MRS supplemented with L-cysteine (0.5 g/L) (Sigma, St. Louis, USA) (MRScys) for isolation of lactic acid bacteria, MacConkey (MCK; BioMérieux, Marcy l’Etoile, France) for isolation of *Enterobacteriaceae*, Baird Parker (BP, BioMérieux) for isolation of staphyloccoci, Sabouraud Dextrose Chloramphenicol (SDC, BioMérieux) for isolation of yeasts, and Brain Heart Infusion (BHI, Oxoid), Wilkins-Chalgren (WC, Oxoid) and Columbia Nadilixic Acid Agar (CNA, BioMérieux) as general media for isolation of other bacterial groups. Plates were aerobically incubated at 37°C for up to 48 h, with the exception of SDC plates, which were incubated at 32°C for 96 h, and WC and MRScys plates that were anaerobically incubated (85% nitrogen, 10% hydrogen, 5% carbon dioxide) in an anaerobic workstation (Mini-MACS Don Whitley Scientific Limited, Shipley, UK) at 37°C for 48 h. Bacterial counts were recorded as the colony forming units (CFU)/g of meconium or feces and transformed to log_10_ values before statistical analysis.

### Bacterial Genotyping and Identification

After bacterial counting, approximately 670 isolates were selected, including at least one representative of each colony morphology type. These isolates were grown in BHI or MRS broth and stored at −80°C in the presence of glycerol (30%, v/v). Isolates were analyzed by optical microscopy to determine cell morphology and Gram-staining reaction, and tested for oxidase, catalase and coagulase activities. Subsequently, they were submitted to Randomly Amplified Polymorphic DNA (RAPD) polymerase chain reaction (PCR) analysis to discard duplicate isolates from the same sample. RAPD profiles were obtained using primer OPL5 (5′-ACGCAGGCAC-3′) [29]. Computer assisted analysis was performed with InfoQuest FP software (Bio-Rad Laboratories, Inc., Hercules, CA). Cluster analysis of RAPD pattern profiles was performed using the UPGMA method based on the Dice similarity coefficient.

Those isolates that displayed identical RAPD profile and had been obtained from the same sample were identified by species-specific PCR or 16S rRNA PCR sequencing. Initially, the isolates that, on the basis of preliminary tests, seemed to belong to the Genus *Staphylococcus* were identified as *Staphylococcus epidermidis*, *Staphylococcus aureus* or *Staphylococcus hominis* by a multiplex PCR method based on the *dnaJ* genes with primers J-StGen (5′-TGGCCAAAAGAGACTATTATGA-3′), J-StEpi (5′-CCACCAAAGCCTTGACTT-3′), JStAur (5′-GGATCTCTTTGTCTGCCG-3′) and JStHom (5′-TTGACCACTACCCTCACAC-3′) [30].

On the other hand, most of the isolates that seemed to belong to the Genus *Enterococcus* were identified by PCR species-specific detection of enterococcal *ddl* genes, which encode D-alanine: D-alanine ligases, following the protocol described by Dutka-Malen et al. [31]. For this purpose, four primers were used: E1 (5′-TCAAGTACAGTTAGTCTT-3′), E2 (5′-ACGATTCAAAGCTAACTG-3′), F1 (5′-GCAAGGCTTCTTAGAGA-3′) and F2 (5′-CATCGTGTAAGCTAACTTC-3′). The first pair (E1 and E2) specifically detects *Enterococcus faecium* strains, while the second (F1 and F2) is specific for *Enterococcus faecalis*.

Identification of other bacterial species was performed by 16S rRNA PCR sequencing (ABI Prism 3730, Applied Biosystems) using primers plb16 (5′-AGAGTTTGATCCTGGCTCAG-3′) and mlb16 (5′-GGCTGCTGGCACGTAGTTAG-3′) [32]. The resulting sequences with an average size of approximately 550 bp, were used to search sequences deposited in the EMBL database using BLAST algorithm and the identity of the isolates was determined on the basis of the highest scores (>98%).

### DNA Extraction from Meconium and Feces

Samples were thawed at room temperature and DNA was extracted from 0.1 g of meconium or feces, previously resuspended in 0.5 mL of extraction buffer (200 mM Tris–HCl pH 7.5, 0.5% SDS, 25 mM EDTA, 250 mM NaCl, 20 mg/mL lysozyme, 5 µg/mL lysostaphin) and 0.3 mL of 3 M Na acetate. Then, mechanical lysis was performed by three times of bead-beating with 0.1 mm diameter zirconia/silica beads (Sigma) using a FastPrep disruptor (QBioGene, Irvine, CA, USA) at a speed setting of 6.0 m/s for 30 s. The lysate solution was treated with 0.1 mg/mL of proteinase K (Sigma), and incubated for 30 min at 37°C. Following incubation, 0.1 mL of 1.5 M NaCl was added to the lysate and mixed. After incubation for 5 min at room temperature, the mixture was centrifuged at 16,000 ×*g* to pellet the insoluble cell debris. The supernatant was transferred into a new tube and extracted twice with an equal volume of phenol/chloroform/isoamyl-alcohol (25∶24:1) (Sigma). The DNA was precipitated by adding 0.6 volumes of isopropanol (Sigma) and incubating at −20°C for 1 h. The DNA was pelleted, washed with 70% ethanol, allowed to air dry, and finally resuspended in TE buffer. The DNA yield was measured using a NanoDrop® ND-1000 UV spectrophotometer (Nano-Drop Technologies, Wilmington, DE).

### PCR Amplification and Denaturing Gradient Gel Electrophoresis (PCR-DGGE)

DGGE of PCR-amplified 16 s rRNA gene fragments was used to initially compare overall bacterial diversity in meconium and feces (3^rd^ week) from 5 infants. To this purpose, primers U968-GC-f (5′-CGCCCGGGGCGCGCCCCGGGCGGGGCGGGGGCACGGGGGGAACGCGAAGAACCTTAC-3′) and L1401-r (5′-CGGTGTGTACAAGACCC-3′) [33], [34] were used to amplify the V6 to V8 regions of the 16S rRNA gene. Then, amplicons were separated by DGGE [35] using a DCode System (Bio-Rad Laboratories). The DGGE profiles were digitally normalized by comparison with a standard pattern using InfoQuest FP software (Bio-Rad Laboratories). Cluster analysis of DGGE profiles was performed using the Neighbor Joining method based on the DICE similarity coefficient.

### Human Intestinal Tract Chip (HITChip) Analysis

The HITChip microarray consists of over 4,800 oligonucleotide probes targeting the V1 and V6 hypervariable regions of the 16S rRNA gene from 1,132 phylotypes, spotted in duplo on custom 8×15 K format arrays (Agilent Technologies, Palo Alto, CA, USA). The array probes were organized based on their 16S rRNA sequences on three levels of phylogeny, as described before [36]. The HITChip signal intensity was analyzed using the following phylogenetic assignment levels: 1) the phylum-level, with the specification of *Firmicutes* down to *Clostridium* clusters and other classes, creating altogether 23 groups; 2) the genus-like level, including 131 groups of sequences with ≥90% sequence identity, and 3) the phylotype (species-like) level with 1,038 distinct phylotypes with ≥98% sequence similarity to cultured species or clones corresponding to uncultured microorganisms. Genus-level taxa with ≥90% sequence identity distributed over multiple genera are termed “*et rel*.”.

All the steps for the HITChip microarray analysis, including PCR amplification of 16S rRNA genes, RNA production and labeling, hybridization and data extraction, were performed basically as described by Rajilic-Stojanovic et al. [34]. Briefly, full-length 16S rRNA gene was amplified using primers T7prom-Bact-27-for (5′-TGAATTGTAATACGACTCACTATAGGGGTTTGATCCTGGCTCAG-3′) and Uni-1492-rev (5′-CGGCTACCTTGTTACGAC-3′), which ensures the introduction of a T7 promoter sequence at the 5′ terminus of the rRNA gene amplicon. Then, the PCR products were purified by using the High Pure PCR Product Purification kit (Roche, Mannheim, Germany), according to the manufacturer’s instructions. *In vitro* transcription of the T7 promoter-carrying 16S rRNA genes was performed using the Riboprobe System (Promega, Madison, WI, USA) while amino-allyl-modified nucleotides were coupled with CyDye using the Post-Labeling Reactive Dye (Amersham Biosciences, Little Chalfont, UK).

Data were extracted from the microarray images using the Agilent Feature Extraction software, version 9.1 (http://www.agilent.com), subsequently normalized, and further analyzed using a set of R-based scripts (http://r-project.org) in combination with a custom-designed relational database that runs under the MySQL database management system (http://www.mysql.com; [34]). Hierarchical clustering of probe profiles was carried out using the Pearson distance and Ward’s minimum variance method. Normalized hybridization signals for 23 Level 1 groups and 131 Level 2 groups are available in Table S1 and Table S2 respectively.

### Statistical Analysis

Quantitative data were expressed as the mean and 95% confidence interval (CI) of the mean or, when they were not normally distributed, as the median and interquartile range (IQR). The richness and diversity of the preterm infants’ meconium and fecal microbiota were determined by calculating the Shannon-Weaver diversity index, which takes into account the number and evenness of the bacterial species. Fisher’s exact test and the Freeman-Halton extension of the Fisher exact probability test for a 2×3 contingency tables were used to compare proportions. Friedman’s non-parametric repeated measures comparisons and paired samples *t*-tests were applied to determine differences between the bacterial counts of each identified microbial group or the hybridization signal intensities of genus-like bacterial groups across time. Principal component analysis (PCA) was applied to the dataset gathering the microbiological profile of all meconium and feces samples obtained after culture methods, to group samples according to their characteristics. Differences were considered significant at *P*<0.05. Statgraphics Centurion XVI version 16.1.15 (Statpoint Technologies Inc., Virginia, USA) and R 2.13.2 (R project, Statistical Software) software were used to carry out the analyses cited above.

## Results

### Characteristics of the Infants

The 14 infants enrolled in this study had a mean gestational age of 28 weeks (ranging from 24 to 32 weeks), a mean birth weight of 1,288 g (ranging from 600 to 2,190 g) (Table 1). Half of the infants (n = 7) were born by Cesarean section, all of them, except one, received antibacterial prophylaxis at least for the first 3 days of life, and half of them needed mechanical ventilation (Table 2). Infants were fed either with their own mother’s breast milk, donor milk and/or preterm formula by nasogastric feeding tube for, at least, 18 days after delivery. The time required for spontaneous delivery of the first meconium oscillated between the first minutes to day 5 after birth. The main characteristics of the infants are presented in Tables 1 and 2.

### Culture Analysis of the Meconium and Fecal Samples

**Figure 1:**
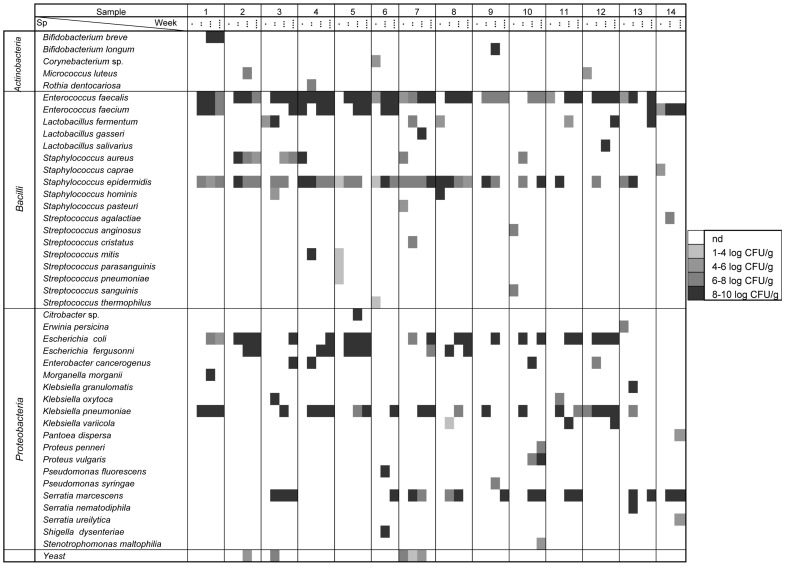
Counts of microbial species isolated and identified by species-specific PCR or 16S rRNA sequencing PCR. Forty-four different species were isolated and identified in some or all the samples analyzed (meconium (·), 1^st^ week (··), 2^nd^ week (···) and 3^rd^ week (····) fecal samples). Concentrations of the identified species are represented in a grey scale from non detected (nd) in white to 8–10 log_10_ CFU/mL in black.

A total of 14 meconium and 39 fecal samples were collected weekly during the first three weeks of life; on average, 3.8 samples per infant were analyzed (Table 1). Globally, inoculation of suitable dilutions of all the samples led to bacterial growth on the culture media tested, with the exception of three meconium samples (infants 1, 2, and 9) (Figure 1).

**Figure 2:**
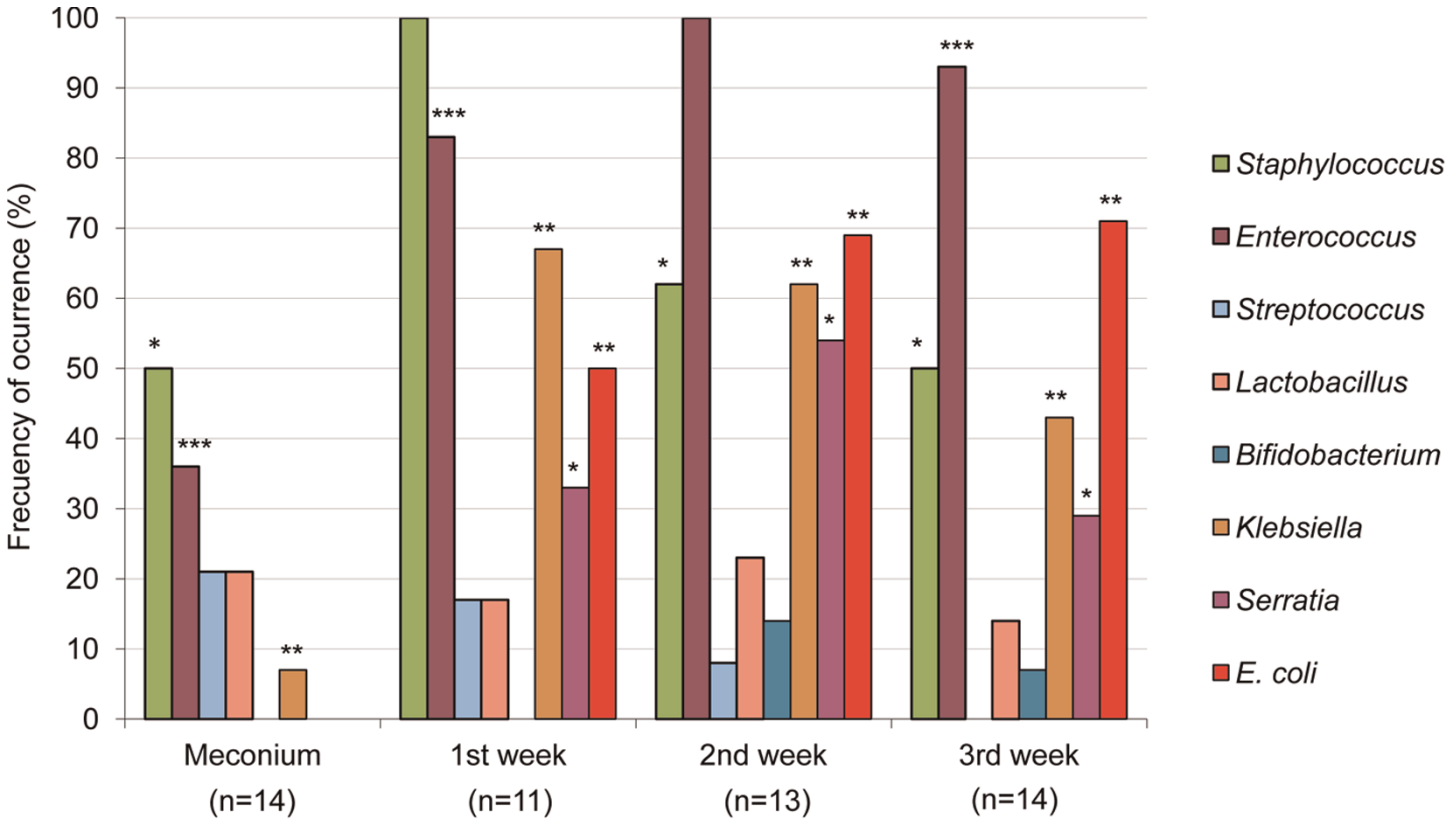
Cultivable bacteria in meconium and fecal samples analyzed in this study. Frequency of occurrence is the percentage of samples in which a particular bacterial genus was detected at each sampling time (meconium, 1^st^ week, 2^nd^ week and 3^rd^ week feces). The total number of weekly samples analyzed is indicated under each sampling time. Results of the Fisher exact probability test (Freeman-Halton extension for a 2×4 contingency table) are shown by asterisks. * = *P*<0.05; ** = *P*<0.01; *** = *P*<0.001.

Identification of the isolates obtained from the different growth media revealed that the most abundant genera in all the tested samples were *Staphylococcus* and *Enterococcus*, which were detected in 77% and 64% of the samples, respectively. However, the distribution of these two genera throughout the sampling times was different (Figure 2). Staphylococci predominated in meconium and 1^st^ week’s fecal samples, being present in 50% and 100% of the samples, respectively, with *S. epidermidis* and *S. aureus* as the most abundant species. Other staphylococcal species, such as *Staphylococcus caprae*, *S. hominis* and *Staphylococcus pasteuri*, were occasionally isolated from meconium or 1^st^ week’s fecal samples (Figure 1). In contrast, *Enterococcus* was the predominant bacterial genus in the 2^nd^ and 3^rd^ week’s fecal samples (100% and 93%, respectively) (Figure 2). *E. faecalis* and *E. faecium* could be isolated from 13 and 7 fecal samples, respectively (Figure 1).

Interestingly, Streptococcus species, such as Streptococcus anginosus, Streptococcus mitis, Streptococcus parasanguinis, Streptococcus pneumoniae, Streptococcus sanguinis and Streptococcus thermophilus, were mainly isolated from meconium samples (Figure 1). The presence of lactobacilli and bifidobacteria was scarce among the isolates obtained on MRScys agar plates. Lactobacillus fermentum was isolated from 6 samples while Lactobacillus gasseri, Lactobacillus salivarius, Bifidobacterium breve and Bifidobacterium longum could only be isolated from the feces of one infant each (Figure 1). In contrast to streptococci, Gram-negative bacteria, such as E. coli, Escherichia fergusonii, Klebsiella pneumoniae and Serratia marcescens, were isolated exclusively from 1^st^, 2^nd^ and/or 3^rd^ week’s fecal samples (Figure 1).

Occasionally, other Gram-positive (belonging to the genera *Corynebacterium*, *Micrococcus* and *Rothia*), and Gram-negative bacteria (belonging to the genera *Citrobacter*, *Enterobacter*, *Erwinia*, *Morganella*, *Pantoea*, *Proteus*, *Pseudomonas*, *Shigella* and *Stenotrophomonas*), as well as some yeasts, were also isolated from the meconium and fecal samples analyzed in this study (Table 3, Figure 1).

**Table 3:** Microbial groups isolated from meconium and feces of preterm neonates in this study.

	Meconium (N = 14)		1^st^ week feces (N = 12)		2^nd^ week feces (N = 13)		3^rd^ week feces (N = 14)		
Microorganism	n	Mean (95% CI)*	n	Mean (95% CI)	n	Mean (95% CI)	n	Mean (95% CI)	*P*-value**
*Staphylococcus*	7	6.48 (3.95; 9.01)	12	7.84 (7.38; 8.31)	8	7.05 (6.26; 7.83)	7	7.22 (6.29; 8.15)	0.037
*Enterococcus*	5	6.15 (3.64; 8.66)	10	8.73 (8.28; 9.18)	13	8.63 (8.13; 9.14)	13	8.26 (7.41; 9.11)	0.003
*Streptococcus*	3	4.77 (0.07; 9.46)	2	7.86	1	6.40	0		0.236
*Lactobacillus*	3	5.27 (2.83; 7.70)	2	7.52	4	7.19 (2.48; 11.90)	2	6.23	0.884
*Bifidobacterium*	0		0		2	9.12	1	9.53	0.145
Other G+	2	5.20	1	7.70	1	7.70	0		0.625
*Klebsiella*	1	7.27	8	9.18 (8.80; 9.55)	8	8.95 (8.13; 9.78)	6	8.72 (8.11; 9.32)	0.004
*Serratia*	0		4	8.73 (6.82; 10.64)	7	9.16 (8.80; 9.53)	4	9.22 (7.80; 10.63)	0.009
*E. coli*	0		6	8.91 (7.89; 9.92)	9	9.05 (8.55; 9.55)	10	8.75 (7.97; 9.53)	0.003
Other G-	1	6.38	1	9.22	5	8.76 (7.33; 10.18)	4	8.40 (5.51; 11.30)	0.118
Yeast	1	6.08	1	7.70	2	5.20	0		0.603

* Mean bacterial counts were expressed as log_10_ CFU/mL.

** Friedman’s tests.

N, total number of samples.

n, number of samples where a specific microbial group was detected.

Other Gram-positive bacteria included the following genera: *Corynebacterium*, *Micrococcus* and *Rothia*.

Other Gram-negative bacteria included the following genera: *Citrobacter, Enterobacter, Erwinia, Morganella, Pantoea, Proteus, Pseudomonas, Shigella* and *Stenotrophomonas.*

Potential associations between demographic and clinical parameters and isolation of the different genera during at least two weeks in fecal samples were assessed using Fisher’s tests. The isolation of the genera *Serratia* seemed to be strongly influenced by demographic or clinical variables related to prematurity. In fact, the presence of *Serratia* was significantly more frequent when the gestational age was <30 weeks (P = 0.020), in longer hospital stay (>35 days; P = 0.027) and in prolonged antibiotherapy treatment (>3 days; P = 0.002). Similarly, samples from infants that required mechanical ventilation showed a higher frequency of *Serratia* (P = 0.050). The isolation of other genera or bacterial groups was not affected by any qualitative variable (Table S3).

Total bacterial counts found when meconium samples were cultured in general media, such as BHI and WC, ranged from 3.65 to 9.85 log_10_ CFU/g whereas in feces samples oscillated between 6.70 and 10.09 log_10_ CFU/g. Mean *Staphylococcus* counts in fecal samples were approximately 1 log_10_ CFU/g higher than in the meconium ones while bacterial counts of other genera, such as *Enterococcus*, *Streptococcus*, *Lactobacillus* or *Klebsiella*, were at least 2 log CFU/g higher in feces than in meconium; however, the differences were statistically-significant only for the genera *Staphylococcus*, *Enterococcus, Klebsiella, Serratia* and *E. coli* (Table 3).

**Figure 3:**
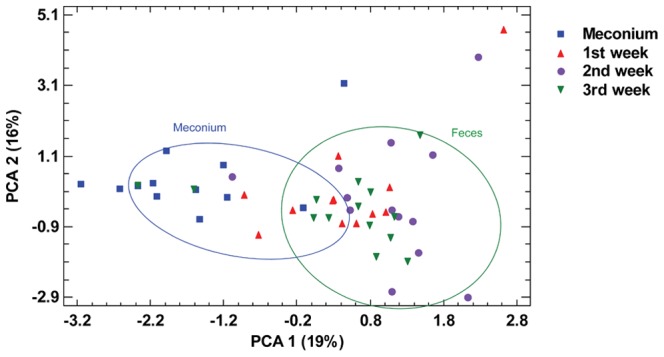
Principal component analysis (PCA) of microbiological profiles of cultivable bacteria. PCA was performed based on the bacterial counts of cultivable bacteria detected at each sampling time: meconium, 1^st^, 2^nd^ and 3^rd^ week feces.

To visualize if differences in microbiological profiles of meconium and fecal samples analyzed in this study existed, principal component analysis (PCA) was applied to the whole dataset of bacterial counts. In the score plot containing the first and second PCs, which account for 36% of total variance, it was observed that meconium samples clustered apart from fecal samples, particularly along the PC1 dimension (Figure 3).

### PCR-DGGE Analysis

**Figure 4:**
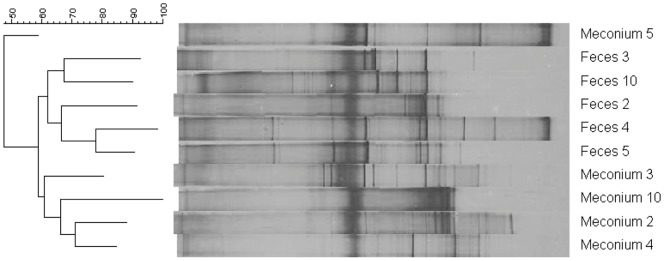
Cluster analysis of DGGE profiles. The analysis was performed using the Neighbor Joining method based on the Dice similarity coefficient in meconium and 3^rd^ week feces from infants 2, 3, 4, 5, and 10.

Meconium and 3^rd^ week’s fecal samples from 5 infants were submitted to PCR-DGGE profiling. The number of major bands in individual infants ranged from 3 to 8 dominant bands. Visual comparison between the DGGE patterns revealed distinctive differences between meconium and fecal samples, being the diversity higher among the fecal ones. DGGE profiles were analyzed by the Neighbor Joining method based on the Dice similarity coefficient. Globally, the profiles clustered in two groups, one including those corresponding to the meconium samples while the second comprised those obtained from 3^rd^ week’s feces. The only exception was the meconium sample collected from infant 5 which clustered with fecal samples. DGGE analyses also revealed a high degree of inter-individual variability among the samples; in fact, similarity values were low, ranging from 50% to 75% (Figure 4).

### HITChip Analysis

**Figure 5:**
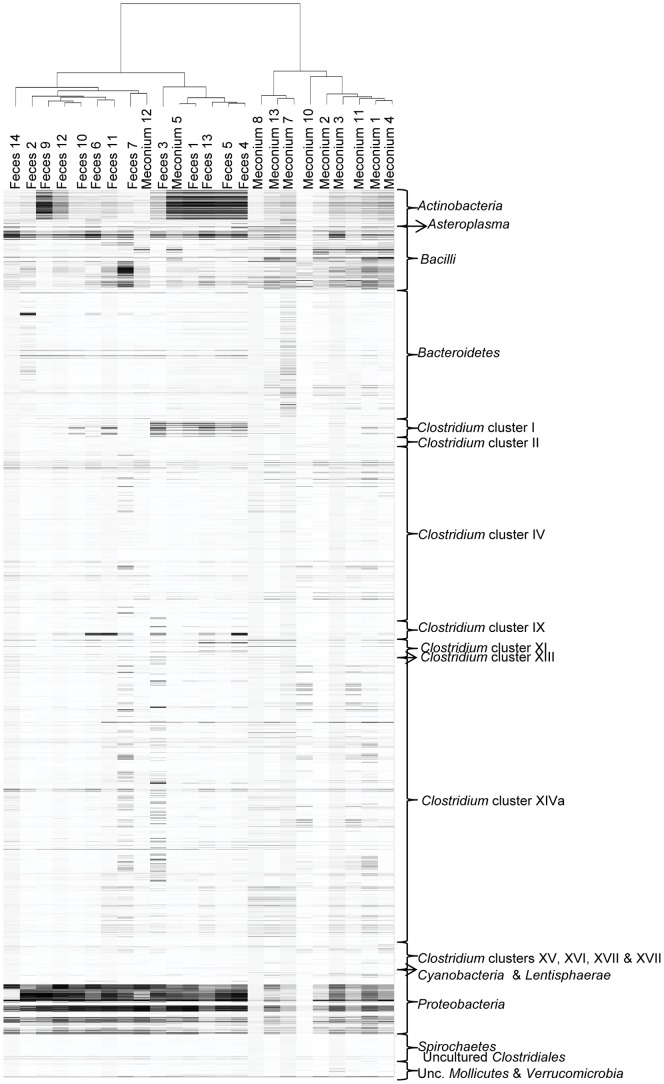
Phylogenetic fingerprints of the meconium and 3^rd^ week fecal microbiota of 14 preterm infants. The highest phylogenetic level of specificity of probes is described on the right side of the figure. Darkness of the spot corresponds to the bacterial abundance in the sample. Pearson correlation and Ward’s clustering method were used.

The microarray datasets of 11 meconium and 13 fecal samples collected in the 3^rd^ week were acquired and hierarchically clustered in a heat map based on the signal intensity of the 3,699 distinct HITChip oligonucleotide probes (Figure 5). Meconium samples (with the exception of those corresponding to infants 5 and 12) and 3^rd^ week’s fecal samples clustered significantly according to the type of sample (meconium *versus* feces).

**Figure 6:**
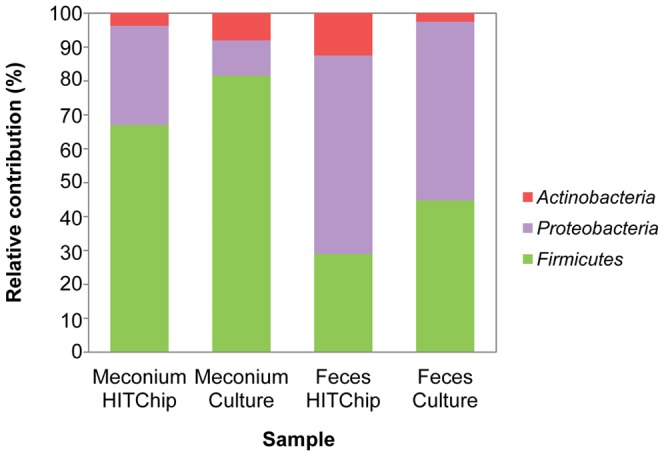
Phyla in the microbiota of meconium and feces analyzed by HITChip and culture-based techniques. The relative contribution of the phyla to the microbiota of meconium and 3^rd^ week fecal samples of fourteen infants detected by culture techniques is shown. In the figure, data for these phyla obtained by HITChip is compared.

**Figure 7:**
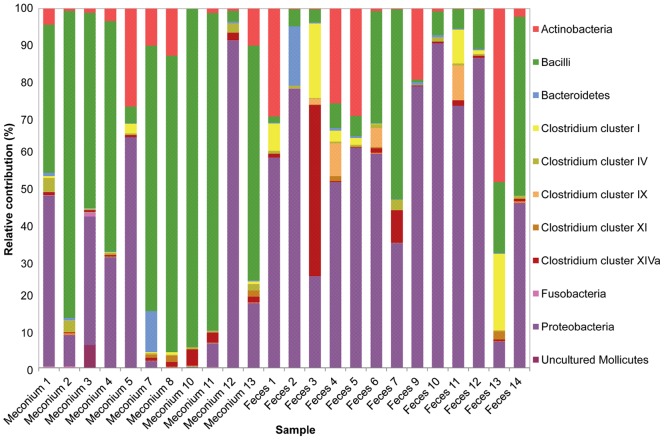
Relative contribution of the phylum/order-like phylogroups to the microbiota of studied infants assessed by HITChip. Phylum/order-like phylogroups detected in meconium and 3^rd^ week fecal samples by HITChip are presented. Only phylum/order-like phylogroups that contributed for at least 0.5% to a given profile are shown.

The relative contributions of the major phyla were assessed by calculating the percentages of the phylum/order taxa in the meconium and 3^rd^ week fecal microbiota. Globally, *Firmicutes* was the most abundant phylum in meconium samples, accounting for approximately 63.4% (95% CI: 42.2%–84.6%) of the signals, followed by *Proteobacteria* (27.7%; 95% CI: 7.61%–47.7%) and *Actinobacteria* (3.5%; IQR: 0.63–10.3) (Figure 6). In contrast, *Proteobacteria* was the dominant phylum in the 3^rd^ week’s fecal samples (57.6%; 95% CI %: 42.8–72.5), followed by *Firmicutes* (28.4%; CI 95%: 13.8%–43.0%) and *Actinobacteria* (12.2%; 95% CI: 2.2%–22.2%). The relative frequency of the three cited phyla differed significantly between the meconium and the fecal samples (Chi-square test; P = 0.021, P = 0.052, and P≤0.001 for *Firmicutes*, *Proteobacteria*, and *Actinobacteria*, respectively). *Bacteroidetes* represented approximately 1% (IQR: 0.1%–0.68%) of the signals in both types of samples (Figure 5 and 7).

Among *Firmicutes*, those belonging to the class *Bacilli* were the most abundant both in meconium (59.8%; 95% CI: 38.4%–81.1%) and 3^rd^ week feces (15.4%; 95% CI: 4.5%–26.3%) samples. However, *Firmicutes* diversity was larger in the fecal samples since other groups were also detected at a relatively high percentage, including *Clostridium* cluster I (5.0%; 95% CI: 0.3%–9.7%), *Clostridium* cluster XIVa (4.9%; IQR: 0.3%–1.3%), and *Clostridium* cluster IX (2.0%; IQR: 0.0%–1.6%) (Figure 7). Fecal samples from infants 3 and 13 showed a different microbial profile with a higher abundance of *Clostridium* clusters and *Actinobacteria*, respectively (Figure 7).

On a lower taxonomic level, the comparison of the 131 hybridization signals, corresponding to the genus-like bacterial groups (level 2), obtained from meconium and fecal samples showed that 38 phylogenetic groups differed significantly between both types of samples. Among them 20 phylogenetic groups contributed for at least more than 0.5% to the microbial profile of each sample (Table 4). The presence of genus-like groups such as *Propionibacterium*, *Lactobacillus plantarum et rel*., *Streptococcus intermedius et rel*. and *Str. mitis et rel*. decreased significantly from meconium to fecal samples. On the other hand, there was a significant increase in the abundance of groups such as *Bacteroides splachnicus et rel*., *Enterococcus*, *Clostridia*, *Veillonella*, *Clostridium difficile et rel*., *E. coli et rel*., *K. pneumoniae et rel*., *Pseudomonas*, *Serratia* and *Yersinia et rel*. groups from meconium to the fecal samples (Table 4). Among the phylogenetic groups contributing ≥0.1% to the fecal profiles, *Enterobacter aerogenes et rel.*, *Enterococcus*, *E. coli et rel*., *Haemophilus*, *K. pneumoniae et rel., Pseudomonas, Serratia, Vibrio* and *Yersinia et rel.* were detected in all the fecal samples, adding up to approximately 55% of the total hybridization signals in ten of them. In contrast, only two phylotype groups, *L. plantarum et rel.* and *Str. mitis et rel*., formed a core microbiome in meconium samples, while there was a great inter-individual variability for the rest of phylotypes (data not shown).

**Table 4:** Abundance of genus-like bacterial groups detected in meconium and 3^rd^ week feces using the HITChip microarray.

Phylum/order	Genus-like phylogenetic group¥	Meconium samplesMean (95% CI)¤	3^rd^ week fecal samplesMean (95% CI)	*P*-value*
*Actinobacteria*	*Bifidobacterium*	3.28 (2.55; 4.01)	4.07 (3.21; 4.93)	0.140
	*Micrococcaceae*	3.06 (2.21; 3.92)	2.97 (2.59; 3.35)	0.957
	*Propionibacterium*	3.63 (3.16; 4.11)	2.27 (1.97–2.77)**	0.004
*Bacteroidetes*	*Bacteroides splachnicus et rel.*	2.38 (1.83; 2.92)	3.48 (3.15; 3.82)	0.005
	*Prevotella tannerae et rel.*	2.06 (1.09; 3.04)	1.22 (0.64; 1.81)	0.040
*Bacilli*	*Aerococcus*	2.87 (2.06; 3.69)	3.36 (3.01; 3.72)	0.165
	*Bacillus*	3.53 (2.94; 4.11)	3.22 (2.87; 3.57)	0.270
	*Enterococcus*	3.98 (3.23; 4.72)	4.90 (4.62; 5.18)	0.025
	*Granulicatella*	2.76 (2.14; 3.38)	3.83 (3.50; 4.16)	0.029
	*Lactobacillus gasseri et rel.*	3.03 (2.54; 3.51)	3.43 (3.01; 3.85)	0.449
	*Lactobacillus plantarum et rel.*	4.43 (3.71; 5.15)	2.38 (2.05; 2.71)	0.000
	*Lactobacillus salivarius et rel.*	3.18 (2.46; 3.91)	3.56 (3.26; 3.86)	0.468
	*Lactococcus*	3.23 (2.44; 4.02)	1.63 (0.54; 2.71)	0.065
	*Staphylococcus*	3.79 (2.97; 4.61)	3.08 (2.60; 3.56)	0.182
	*Streptococcus bovis et rel.*	3.26 (2.90; 3.63)	2.76 (2.39–3.09)**	0.361
	*Streptococcus intermedius et rel.*	3.72 (3.12; 4.32)	2.60 (1.91; 3.28)	0.018
	*Streptococcus mitis et rel.*	4.25 (3.71; 4.79)	2.95 (2.33; 3.58)	0.006
	*Weissella et rel.*	2.40 (1.54; 3.25)	2.83 (2.20; 3.47)	0.263
*Clostridium* cluster I	*Clostridia*	2.88 (2.18; 3.57)	3.80 (2.93; 4.67)	0.013
*Clostridium* cluster IV	*Clostridium leptum et rel.*	2.86 (2.36; 3.35)	2.99 (2.52; 3.46)	0.717
	*Clostridium orbiscindens et rel.*	2.91 (2.36; 3.45)	1.45 (1.09–1.72)	0.089
	*Sporobacter termitidis et rel.*	3.03 (2.35; 3.71)	3.39 (2.97; 3.82)	0.203
*Clostridium* cluster IX	*Veillonella*	1.08 (−0.01; 2.17)	2.90 (1.95; 3.85)	0.034
*Clostridium* cluster XI	*Clostridium difficile et rel.*	2.79 (2.21; 3.36)	3.06 (2.57; 3.54)	0.013
*Clostridium* cluster XIVa	*Anaerostipes caccae et rel.*	2.45 (1.93; 2.96)	2.43 (2.12; 2.73)	0.961
	*Bryantella formatexigens et rel.*	2.29 (1.80; 2.80)	2.16 (1.42; 2.89)	0.985
	*Butyrivibrio crossotus et rel.*	2.39 (1.85; 2.94)	1.55 (1.34–1.68)**	0.111
	*Ruminococcus obeum et rel.*	2.47 (1.94; 3.00)	1.92 (1.49; 2.34)	0.114
*Proteobacteria*	*Anaerobiospirillum*	2.13 (1.66; 2.61)	3.93 (3.47; 4.40)	0.001
	*Aquabacterium*	2.86 (2.15; 3.58)	0.28 (−0.86; 1.42)	0.001
	*Burkholderia*	3.14 (2.27; 4.00)	1.69 (1.15; 2.23)	0.074
	*Enterobacter aerogenes et rel.*	3.69 (2.79; 4.59)	4.78 (4.46; 5.10)	0.096
	*Escherichia coli et rel.*	3.63 (2.54; 4.72)	5.47 (5.18; 5.75)	0.043
	*Haemophilus*	3.31 (2.49; 4.12)	4.25 (4.05; 4.46)	0.026
	*Klebsiella pneumoniae et rel.*	3.90 (2.88; 4.93)	5.33 (5.06–5.38)**	0.034
	*Leminorella*	2.66 (2.04; 3.29)	3.08 (2.56; 3.60)	0.822
	*Proteus et rel.*	3.05 (2.23; 3.87)	3.89 (3.61–4.33)**	0.259
	*Pseudomonas*	3.31 (2.49; 4.12)	4.19 (3.98; 4.41)	0.038
	*Serratia*	3.01 (2.18; 3.83)	3.95 (3.74; 4.16)	0.031
	*Sutterella wadsworthia et rel.*	3.57 (2.99; 4.13)	3.38 (3.22–3.45)**	0.864
	*Vibrio*	3.45 (2.55; 4.34)	4.50 (4.37–4.52)**	0.303
	*Xanthomonadaceae*	2.81 (1.93; 3.69)	2.50 (2.04; 2.96)	0.001
	*Yersinia et rel.*	3.51 (2.51; 4.50)	4.91 (4.65; 5.18)	0.042

¥ The genus-like phylogenetic groups shown contributed, at least, 0.5% to the microbial profile of a given sample.

* Kruskal-Wallis tests.

¤ Mean (95% CI) of the log-transformed signal intensities.

** Median (IQR) of the log-transformed signal intensities.

Significant changes in abundance of genus-like bacterial groups underlined (*P* values <0.05).

The detailed analysis of signal intensities of species-like taxa (level 3) showed an average of 441 phylotype-like taxa in the meconium samples and 406 of these in the fecal samples, representing 42% and 39% of the total number of the phylotype-like taxa that were above the signal threshold, respectively. The intensity of the hybridization signals obtained with the oligonucleotide probes corresponding to *L. fermentum*, *Lactobacillus reuteri*, *S. epidermidis*, *Streptococcus viridans* and uncultured *Streptococcus* sp. NB4D2 was particularly strong among the meconium samples, while phylotype probes corresponding to *Enterobacter cloacae*, *E. faecalis*, *E. coli*, *Hafnia alvei*, *K. pneumoniae* subsp. *ozaenae*, *Serratia liquefaciens* and *Shigella dysenteriae* led to more intense hybridization intensities among the fecal ones (Table S4). Proteobacterial phylotype-like taxa were mainly detected in fecal samples and, also, in the meconium samples obtained from infants 5 and 12 (Table S5). With respect to the *Lactobacillus* and *Lactococcus* phylotype-like taxa, a total of 15 different phylotypes were detected, being the meconium obtained from of infants 1, 2, 4 and 11 the ones that showed the highest diversity (Table S6). In meconium from infant 2, *Lactobacillus* phylotype-like taxa represented 82% of the total hybridization signals while those of *L. reuteri* and *Lactobacillus vaginalis* contributed with 45% and 27% to the total hybridization signals, respectively (Table S6). Bifidobacterial phylotype-like taxa were mainly detected in one meconium (infant 5) and five fecal samples (infants 1, 4, 5, 9 and 13) (Table S7). Among the 12 bifidobacteria phylotype-like taxa detected in this study, those of *B. breve*, *Bifidobacterium catenolatum* and *Bifidobacterium infantis* were the most abundant. In the fecal sample of infant 13, 44% of the hybridization signals corresponded to bifidobacterial species while, in the rest of fecal samples where they were present, they represented between 19 and 27% of the signal intensities (Table S7). Phylotype-like taxa belonging to the *Clostridium* spp. were infrequent either in meconium or fecal samples, except in the fecal sample of infant 13 where they represented 17% of the total hybridization signal (data not shown).

### Comparison between the Results Obtained by Cultures and HITChip

Globally, there was a good correlation between the principal phylum/orders detected in the samples by the culturing and molecular approach (Figure 6). In general, the class of the *Bacilli* was the predominant bacterial group among meconium samples whereas *Proteobacteria* was the main phylum observed among the fecal ones.

Considering the genus-like taxonomy level, both culture and HITChip techniques showed that *Enterococcus* was a relevant group in the meconium and/or feces of the preterm infants. Other genera, such as *Bifidobacterium, Lactobacillus* or *Streptococcus*, could be detected by both techniques but the detection frequency was lower using the culture-based approach (Tables 3 and 4). In addition, the HITChip technique allowed the detection of other bacterial genera, such as *Lactococcus* or *Clostridium* that could not be isolated in the cultures of the respective samples.

**Figure 8:**
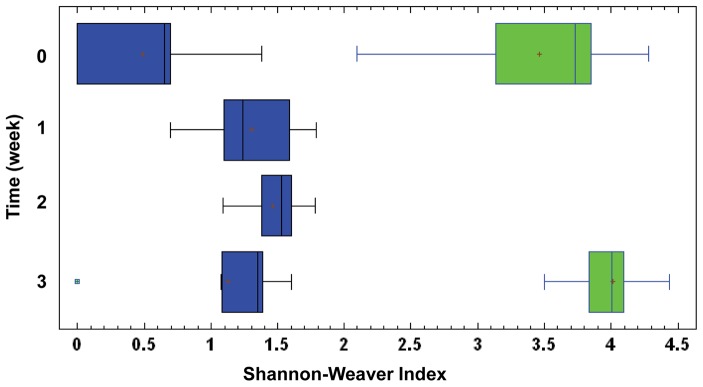
Shannon-Weaver diversity indices from culture and HITChip results. Boxes in blue represent Shannon-Weaver diversity index obtained with culture techniques in meconium (0 week), 1^st^, 2^nd^ and 3^rd^ week feces. Boxes in green represent Shannon-Weaver diversity index obtained with HITChip in meconium (0 week) and 3^rd^ week feces.

Finally, the diversity of the microbial communities obtained by culturing or using the HITChip was also evaluated. The Shannon-Weaver indices obtained with culture techniques were lower than those obtained with the microarray, which indicated a superior sensitivity of the molecular technique (Figure 8). Anyway, both methods revealed higher bacterial diversity in fecal than in meconium samples (Paired t-test; *P* = 0.016 and *P = *0.011, for HITChip and culture techniques, respectively). The Shannon diversity index showed a greater variability among the meconium samples (ranging from 0.00 to 1.38 with culture techniques, and from 2.09 to 4.28 with microarrays) than among the fecal ones (ranging from 1.07 to 1.60 with culture techniques, and from 3.50 to 4.43 with microarrays).

## Discussion

In the present study, the succession of the bacterial species in meconium and feces of preterm infants in their first 3 weeks of life was assessed by culture-based methods, PCR-DGGE and the HITChip microarray. The combination of culture analysis and culture-independent techniques provided highly complementary views of the microbiota present in the analyzed samples. The results obtained by both approaches showed a good correlation although the HITChip method was most sensitive and led to the detection of higher bacterial diversity, probably due to the different recovery/survival after sample thawing and complex nutritional requirements of some species when using culture methods. Other differences between culture- and molecular-based methods could be related to PCR bias, cross hybridization and difficulty of detection if species are present in low levels, in agreement with previous studies [18]. In general, analysis of meconium and feces of preterm infants revealed low species diversity and high inter-individual variability, as previously described [13], [14], [37], [38]. Bacterial diversity in meconium samples was lower than in feces although the diversity indices obtained in both types of samples upon HITChip analysis were higher than those reported in previous studies [13], [14], [16]. *Bacilli* and other *Firmicutes* dominated in the meconium and *Proteobacteria* did in the fecal samples. At the genus level, *Staphylococcus, Streptococcus*, *Enterococcus* and *Lactobacillus* predominated in meconium samples while members of the family Enterobacteriaceae, such as *Escherichia*, *Klebsiella* or *Serratia,* rapidly became dominant in feces, a fact that has been repeatedly reported [11], [12], [13], [14], [15], [16], [38], [39]. In a previous work [27] where meconium samples from term healthy babies born at the same hospital were analyzed, *E. coli* and *Leuconostoc* sp. were detected by culture methods, as it has been described recently by Gosalbes et al [7] with molecular techniques. In contrast, in this work these species had not been isolated by culture methods although a low signal intensity of *E. coli* was detected by HITChip in meconium samples of preterm infants. It should be taken into account that freezing can introduce microbiological biases in culture methods as indicated before.

Premature birth usually results in a delayed and abnormal qualitative pattern of gut colonization, which is often described as aberrant in comparison to that of healthy term ones [5], [6]. This fact seems to affect infant’s health and constitutes a risk factor for the development of gastrointestinal infections, such as necrotizing enterocolitis [21], [40]. In a recent study, where meconium and fecal samples from 6 preterm infants were analyzed by 16S rRNA high throughput pyrosequencing, it was found that gut colonization seemed to follow specific patterns [23]. Subjects who develop sepsis showed a *Proteobacteria* and *Firmicutes* (*Staphylococcus*) predominance. Notably, healthy subjects, who received limited antibiotics (<3 days total) and did not develop sepsis ultimately, exhibited an increase in relative abundance of anaerobes, similarly to more ‘mature’ microbial communities, including *Clostridium*, *Klebsiella* and *Veillonella* [23]. The fecal microbiota of preterm infants is usually dominated by cultivable bacteria that are prevalent in antibiotic-rich hospital environments, such as NICUs. In this study, presence of *Serratia* was strongly associated with several hospital-related parameters, including antibiotherapy and mechanical ventilation. Fear of infections often leads to an early and widespread use of broad-spectrum antibiotics at the NICUs, a strategy that increases the risk of colonization with resistant bacterial strains [41]. The high influence of the environment is in accordance with previous studies that reported a tendency to uniformity in the bacterial communities of preterm infants during their stay at the NICU [16]. Likewise, it has been recently shown that the NICU was a major determinant influencing clostridia colonization of preterm infants and that antibiotic course influenced the levels of colonization [42]. A recent in depth pyrosequencing study examined the gut-associated microbiome of 11 extremely low birth weight infants in the first postnatal month. This study confirmed that Enterobacteriales, *Staphylococcus*, and *Enterococcus* were among the most abundant bacterial taxa in a low-diversity bacterial community that was dominated by types of bacteria known to cause invasive disease in these infants [12].

In preterm neonates, colonization by strict anaerobes seems particularly delayed [13]. Clostridia colonization varies greatly from baby to baby in the timing of their first appearance while *Bacteroides* and bifidobacteria have been seldom isolated from feces of these infants [38], [43], [44]. In this study, bifidobacteria and bifidobacterial DNA could be isolated and detected, albeit at a low frequency. A previous study showed that the birth gestational age had a significant impact on infant colonization by bifidobacteria, which always occurred in children born at a birth gestational age greater than 33 weeks [10]. In fact, some studies have evidenced the relatively low frequency and abundance of bifidobacteria in the fecal microbiota at any age from birth to adulthood [6]. These authors suggested that the emphasis on bifidobacteria in studies and reviews of the infant gastrointestinal microbiota may be out of proportion to its prevalence, abundance, and relevance to health. This aspect should be addressed in future work.

Globally, our results indicate that spontaneously-released meconium of preterm neonates harbors a specific microbiota, different to that of feces obtained after the first week of life. Previous studies have shown the presence of a similar cultivable microbiota in umbilical chord’s blood and hygienically-collected meconium of term and preterm infants, suggesting that fetal gut may not be sterile before delivery and that, therefore, at least a part of the bacteria found in meconium has not a postnatal origin [14], [26], [27].

Probably, such bacteria could reach the fetal gut through *in utero* swallowing of amniotic fluid. Culture-dependent and -independent studies have revealed that there are bacteria in human amniotic fluid without rupture of membranes [24]. Previously, we showed that oral administration of an enterococcal strain to pregnant mice led to their presence in amniotic fluid and meconium obtained by Cesarean section [26]; this fact is not surprising since it has been reported that bacteria of the digestive tract can reach amniotic fluid through the blood stream [45]. Another study carried out in pregnant women focused on the influence of the composition of their oral microbiota in the pregnancy outcome and showed that some bacteria, such as *Actinomyces naselundii*, were associated to lower birth weight and earlier delivery, while others, such as lactobacilli, were linked with a higher birth weight and later delivery date. The results of such study suggested that oral bacteria can enter the uterine environment through the bloodstream and may influence the delivery process [46]. Streptococci and staphylococci seemed to be among the dominant bacteria in the meconium samples and, interestingly, their presence in chorioamnion samples of healthy mothers submitted to caesarean section has been described previously [47]. The potential existence of initial gut colonization during the fetal stage deserves future research since it could have important health implications; as an example, its modulation during pregnancy could help to avoid premature deliveries.

## Supporting Information

Table S1 Normalized hybridization signal for all 23 Level 1 (phylum-like) phylogenetic groups targeted by the HITChip.
(XLSX)

Table S2 Normalized hybridization signal intensity for all 131 Level 2 (genus-like) phylogenetic groups targeted by the HITChip.
(XLSX)

Table S3 Associations between demographic and clinical data and the presence of the isolated genera or microbial group during at least two weeks in the analyzed assessed using Fisher’s tests.
(DOCX)

Table S4 Dominant phylotypes in meconium and 3^rd^ week fecal samples detected by HITChip.
(DOCX)

Table S5 Proteobacterial phylotypes detected in meconium and 3^rd^ week fecal samples using HITChip technique.
(DOCX)

Table S6 Lactobacilli and lactococci phylotypes detected in meconium and 3^rd^ week fecal samples using HITChip technique.
(DOCX)

Table S7 Bifidobacterial phylotypes detected in meconium and 3^rd^ week fecal samples using HITChip technique.
(DOCX)
