# Supplementary material for: Bacterial Diversity in Meconium of Preterm Neonates and Evolution of Their Fecal Microbiota during the First Month of Life
Source: PLoS One. 2013 Jun 28;8(6):e66986. doi: 10.1371/journal.pone.0066986 (PMC3695978; doi:10.1371/journal.pone.0066986)
Supplement: Table S3 — Associations between demographic and clinical data and the presence of the isolated genera or microbial group during at least two weeks in the analyzed assessed using Fisher’s tests. (DOCX) [file pone.0066986.s003.docx]

Table S3. Association between demographic and clinical data and the presence of the isolated genera or microbial group in the analyzed samples.

|  |  | *Staphylococcus* | |  |  | *Enterococcus* |  |  |  | *Streptococcus* |  |  |  |
| --- | --- | --- | --- | --- | --- | --- | --- | --- | --- | --- | --- | --- | --- |
|  |  | Meconium |  | Feces |  | Meconium |  | Feces |  | Meconium |  | Feces |  |
|  |  | Frequency (%)* | *P* value | Frequency (%) | *P* value | Frequency (%) | *P* value | Frequency (%) | *P* value | Frequency (%) | *P* value | Frequency (%) | *P* value |
| Gestational age (wk) | 24-26 (n=4) | 21.43 | 0.790 | 21.43 | 0.760 | 7.14 | 0.253 | 21.43 | 0.250 | 14.29 | 0.407 | 0.00 | 1.000 |
|  | 27-30 (n=4) | 14.29 |  | 14.29 |  | 21.43 |  | 28.57 |  | 0.00 |  | 0.00 |  |
|  | >30 (n=6) | 14.29 |  | 35.71 |  | 7.14 |  | 42.86 |  | 7.14 |  | 0.00 |  |
| Delivery mode | Cesarean section (n=7) | 28.57 | 0.500 | 35.71 | 0.720 | 21.43 | 0.500 | 42.86 | 0.499 | 7.14 | 0.500 | 0.00 | 1.000 |
|  | Vaginal (n=7) | 21.43 |  | 35.71 |  | 14.29 |  | 50.00 |  | 14.29 |  | 0.00 |  |
| Birth weight (g) | <1000 (n=4) | 21.43 | 0.359 | 21.43 | 0.131 | 7.14 | 1.000 | 21.43 | 0.286 | 14.29 | 0.231 | 0.00 | 1.000 |
|  | 1000-1500 (n=5) | 7.14 |  | 14.29 |  | 14.29 |  | 35.71 |  | 0.00 |  | 0.00 |  |
|  | >1500 (n=5) | 21.43 |  | 35.71 |  | 14.29 |  | 35.71 |  | 7.14 |  | 0.00 |  |
| Antibiotherapy (days) | > 3 (n=7) | 35.71 | 0.143 | 35.71 | 0.720 | 21.43 | 0.500 | 42.86 | 0.499 | 14.29 | 0.500 | 0.00 | 1.000 |
|  | < 3 (n=7) | 14.29 |  | 35.71 |  | 14.29 |  | 50.00 |  | 7.14 |  | 0.00 |  |
| Parenteral nutrition (days) | 0 (n=3) | 14.29 | 0.111 | 21.43 | 0.595 | 7.14 | 1.000 | 21.43 | 0.571 | 7.14 | 0.258 | 0.00 | 1.000 |
|  | 0-5 (n=6) | 7.14 |  | 28.57 |  | 14.29 |  | 42.86 |  | 0.00 |  | 0.00 |  |
|  | > 5 (n=5) | 28.57 |  | 21.43 |  | 14.29 |  | 28.57 |  | 14.29 |  | 0.00 |  |
| Nasogastric feeding tube (days) | < 50 (n=9) | 14.29 | 0.296 | 50.00 | 0.454 | 7.14 | 0.238 | 64.29 | 0.357 | 7.14 | 0.615 | 0.00 | 1.000 |
|  | > 50 (n=5) | 35.71 |  | 21.43 |  | 28.57 |  | 28.57 |  | 14.29 |  | 0.00 |  |
| Mechanical ventilation | Yes (n=7) | 35.71 | 0.143 | 42.86 | 0.279 | 21.43 | 0.500 | 42.86 | 0.499 | 14.29 | 0.500 | 0.00 | 1.000 |
|  | No (n=7) | 14.29 |  | 28.57 |  | 14.29 |  | 50.00 |  | 7.14 |  | 0.00 |  |
| Hospital stay (days) | < 35 (n=5) | 14.29 | 0.500 | 28.57 | 0.545 | 7.14 | 0.378 | 35.71 | 0.642 | 7.14 | 0.725 | 0.00 | 1.000 |
|  | > 35 (n=9) | 35.71 |  | 42.86 |  | 28.57 |  | 57.14 |  | 14.29 |  | 0.00 |  |
| Meconium expulsion (h) | < 12 (n=7) | 28.57 | 0.500 | 35.71 | 0.720 | 14.29 | 0.500 | 50.00 | 0.499 | 7.14 | 0.500 | 0.00 | 1.000 |
|  | > 12 (n=7) | 21.43 |  | 35.71 |  | 21.43 |  | 42.86 |  | 14.29 |  | 0.00 |  |
| Type of feeding | Breast-fed (n=7) | 21.43 | 0.500 | 28.57 | 0.279 | 21.43 | 0.500 | 42.86 | 0.499 | 14.29 | 0.500 | 0.00 | 1.000 |
|  | Mixed-fed (n=7) | 28.57 |  | 42.86 |  | 14.29 |  | 50.00 |  | 7.14 |  | 0.00 |  |

Table S3 (continuation)

|  |  | *Lactobacillus* |  |  |  | *Bifidobacterium* | |  |  | Other G+ |  |  |  |
| --- | --- | --- | --- | --- | --- | --- | --- | --- | --- | --- | --- | --- | --- |
|  |  | Meconium |  | Feces |  | Meconium |  | Feces |  | Meconium |  | Feces |  |
|  |  | Frequency (%) | *P* value | Frequency (%) | *P* value | Frequency (%) | *P* value | Frequency (%) | *P* value | Frequency (%) | *P* value | Frequency (%) | *P* value |
| Gestational age (wk) | 24-26 (n=4) | 7.14 | 0.209 | 0.00 | 0.736 | 0.00 | 1.000 | 0.00 | 1.000 | 7.14 | 1.000 | 0.00 | 1.000 |
|  | 27-30 (n=4) | 14.29 |  | 7.14 |  | 0.00 |  | 0.00 |  | 0.00 |  | 0.00 |  |
|  | >30 (n=6) | 0.00 |  | 14.29 |  | 0.00 |  | 7.14 |  | 7.14 |  | 0.00 |  |
| Delivery mode | Cesarean section (n=7) | 14.29 | 0.500 | 7.14 | 0.500 | 0.00 | 1.000 | 0.00 | 0.499 | 7.14 | 0.769 | 0.00 | 1.000 |
|  | Vaginal (n=7) | 7.14 |  | 14.29 |  | 0.00 |  | 7.14 |  | 7.14 |  | 0.00 |  |
| Birth weight (g) | <1000 (n=4) | 7.14 | 1.000 | 0.00 |  | 0.00 | 1.000 | 0.00 | 0.999 | 7.14 | 0.725 | 0.00 | 1.000 |
|  | 1000-1500 (n=5) | 7.14 |  | 7.14 | 0.725 | 0.00 |  | 0.00 |  | 7.14 |  | 0.00 |  |
|  | >1500 (n=5) | 7.14 |  | 14.29 |  | 0.00 |  | 7.14 |  | 0.00 |  | 0.00 |  |
| Antibiotherapy (days) | > 3 (n=7) | 21.43 | 0.096 | 7.14 | 0.500 | 0.00 | 1.000 | 0.00 | 0.499 | 7.14 | 0.769 | 0.00 | 1.000 |
|  | < 3 (n=7) | 0.00 |  | 14.29 |  | 0.00 |  | 7.14 |  | 7.14 |  | 0.00 |  |
| Parenteral nutrition (days) | 0 (n=3) | 0.00 | 0.753 | 7.14 | 0.382 | 0.00 | 1.000 | 0.00 | 0.999 | 0.00 | 1.000 | 0.00 | 1.000 |
|  | 0-5 (n=6) | 14.29 |  | 14.29 |  | 0.00 |  | 7.14 |  | 7.14 |  | 0.00 |  |
|  | > 5 (n=5) | 7.14 |  | 0.00 |  | 0.00 |  | 0.00 |  | 7.14 |  | 0.00 |  |
| Nasogastric feeding tube (days) | < 50 (n=9) | 7.14 | 0.615 | 14.29 | 0.725 | 0.00 | 1.000 | 7.14 | 0.643 | 0.00 | 0.308 | 0.00 | 1.000 |
|  | > 50 (n=5) | 14.29 |  | 7.14 |  | 0.00 |  | 0.00 |  | 14.29 |  | 0.00 |  |
| Mechanical ventilation | Yes (n=7) | 21.43 | 0.096 | 14.29 | 0.500 | 0.00 | 1.000 | 0.00 | 0.499 | 7.14 | 0.769 | 0.00 | 1.000 |
|  | No (n=7) | 0.00 |  | 7.14 |  | 0.00 |  | 7.14 |  | 7.14 |  | 0.00 |  |
| Hospital stay (days) | < 35 (n=5) | 21.43 | 0.231 | 14.29 | 0.275 | 0.00 | 1.000 | 7.14 | 0.357 | 7.14 | 0.604 | 0.00 | 1.000 |
|  | > 35 (n=9) | 0.00 |  | 7.14 |  | 0.00 |  | 0.00 |  | 7.14 |  | 0.00 |  |
| Meconium expulsion (h) | < 12 (n=7) | 7.14 | 0.500 | 14.29 | 0.500 | 0.00 | 1.000 | 7.14 | 0.499 | 7.14 | 0.769 | 0.00 | 1.000 |
|  | > 12 (n=7) | 14.29 |  | 7.14 |  | 0.00 |  | 0.00 |  | 7.14 |  | 0.00 |  |
| Type of feeding | Breast-fed (n=7) | 14.29 | 0.500 | 14.29 | 0.500 | 0.00 | 1.000 | 7.14 | 0.499 | 14.29 | 0.231 | 0.00 | 1.000 |
|  | Mixed-fed (n=7) | 7.14 |  | 7.14 |  | 0.00 |  | 0.00 |  | 0.00 |  | 0.00 |  |

Table S3 (continuation)

|  |  | *Klebsiella* |  |  |  | *Serratia* |  |  |  | *E. coli* |  |  |  |
| --- | --- | --- | --- | --- | --- | --- | --- | --- | --- | --- | --- | --- | --- |
|  |  | Meconium |  | Feces |  | Meconium |  | Feces |  | Meconium |  | Feces |  |
|  |  | Frequency (%) | *P* value | Frequency (%) | *P* value | Frequency (%) | *P* value | Frequency (%) | *P* value | Frequency (%) | *P* value | Frequency (%) | *P* value |
| Gestational age (wk) | 24-26 (n=4) | 0.00 | 1.000 | 14.29 | 0.999 | 0.00 | 1.000 | 21.43 | 0.020 | 0.00 | 1.000 | 14.29 | 0.481 |
|  | 27-30 (n=4) | 0.00 |  | 21.43 |  | 0.00 |  | 21.43 |  | 0.00 |  | 14.29 |  |
|  | >30 (n=6) | 7.14 |  | 28.57 |  | 0.00 |  | 0.00 |  | 0.00 |  | 35.71 |  |
| Delivery mode | Cesarean section (n=7) | 0.00 | 0.500 | 35.71 |  | 0.00 | 1.000 | 21.43 | 0.703 | 0.00 | 1.000 | 35.71 |  |
|  | Vaginal (n=7) | 7.14 |  | 28.57 |  | 0.00 |  | 21.43 |  | 0.00 |  | 28.57 | 0.499 |
| Birth weight (g) | <1000 (n=4) | 0.00 | 0.999 | 14.29 | 0.800 | 0.00 | 1.000 | 21.43 | 0.334 | 0.00 | 1.000 | 14.29 | 0.151 |
|  | 1000-1500 (n=5) | 7.14 |  | 21.43 |  | 0.00 |  | 14.29 |  | 0.00 |  | 14.29 |  |
|  | >1500 (n=5) | 0.00 |  | 28.57 |  | 0.00 |  | 7.14 |  | 0.00 |  | 35.71 |  |
| Antibiotherapy (days) | > 3 (n=7) | 0.00 | 0.500 | 42.86 | 0.499 | 0.00 | 1.000 | 42.86 | 0.002 | 0.00 | 1.000 | 21.43 | 0.133 |
|  | < 3 (n=7) | 7.14 |  | 35.71 |  | 0.00 |  | 0 |  | 0.00 |  | 42.86 |  |
| Parenteral nutrition (days) | 0 (n=3) | 0.00 | 1.000 | 14.29 | 0.413 | 0.00 | 1.000 | 0.00 | 0.112 | 0.00 | 1.000 | 14.29 | 0.413 |
|  | 0-5 (n=6) | 7.14 |  | 35.71 |  | 0.00 |  | 14.29 |  | 0.00 |  | 35.71 |  |
|  | > 5 (n=5) | 0.00 |  | 14.29 |  | 0.00 |  | 28.57 |  | 0.00 |  | 14.29 |  |
| Nasogastric feeding tube (days) | < 50 (n=9) | 0.00 | 0.571 | 50.00 | 0.202 | 0.00 | 1.000 | 14.29 | 0.060 | 0.00 | 1.000 | 50.00 | 0.202 |
|  | > 50 (n=5) | 7.14 |  | 14.29 |  | 0.00 |  | 28.57 |  | 0.00 |  | 14.29 |  |
| Mechanical ventilation | Yes (n=7) | 0.00 | 0.500 | 35.71 | 0.499 | 0.00 | 1.000 | 35.71 | 0.050 | 0.00 | 1.000 | 28.57 | 0.499 |
|  | No (n=7) | 7.14 |  | 28.57 |  | 0.00 |  | 7.14 |  | 0.00 |  | 35.71 |  |
| Hospital stay (days) | < 35 (n=5) | 7.14 | 0.357 | 28.57 | 0.377 | 0.00 | 1.000 | 0.00 | 0.027 | 0.00 | 1.000 | 28.57 | 0.378 |
|  | > 35 (n=9) | 0.00 |  | 35.71 |  | 0.00 |  | 42.86 |  | 0.00 |  | 35.71 |  |
| Meconium expulsion (h) | < 12 (n=7) | 7.14 | 0.500 | 35.71 | 0.499 | 0.00 | 1.000 | 14.29 | 0.296 | 0.00 | 1.000 | 35.71 | 0.499 |
|  | > 12 (n=7) | 0.00 |  | 28.57 |  | 0.00 |  | 28.57 |  | 0.00 |  | 28.57 |  |
| Type of feeding | Breast-fed (n=7) | 7.14 | 0.500 | 0.50 | 0.725 | 0.00 | 1.000 | 14.29 | 0.296 | 0.00 | 1.000 | 42.86 | 0.133 |
|  | Mixed-fed (n=7) | 0.00 |  | 14.29 |  | 0.00 |  | 28.57 |  | 0.00 |  | 21.43 |  |

Table S3 (continuation)

|  |  | Other G- |  |  |  | Yeast |  |  |  |
| --- | --- | --- | --- | --- | --- | --- | --- | --- | --- |
|  |  | Meconium |  | Feces |  | Meconium |  | Feces |  |
|  |  | Frequency (%) | *P* value | Frequency (%) | *P* value | Frequency (%) | *P* value | Frequency (%) | *P* value |
| Gestational age (wk) | 24-26 (n=4) | 7.14 | 0.571 | 0.00 | 1.000 | 0.00 | 0.571 | 7.14 | 0.473 |
|  | 27-30 (n=4) | 0.00 |  | 0.00 |  | 7.14 |  | 7.14 |  |
|  | >30 (n=6) | 0.00 |  | 7.14 |  | 0.00 |  | 0.00 |  |
| Delivery mode | Cesarean section (n=7) | 7.14 | 0.500 | 0.00 | 0.499 | 7.14 | 0.500 | 0.00 | 0.231 |
|  | Vaginal (n=7) | 0.00 |  | 7.14 |  | 0.00 |  | 14.29 |  |
| Birth weight (g) | <1000 (n=4) | 7.14 | 0.286 | 0.00 | 0.999 | 0.00 | 1.000 | 7.14 | 0.725 |
|  | 1000-1500 (n=5) | 0.00 |  | 0.00 |  | 0.00 |  | 0.00 |  |
|  | >1500 (n=5) | 0.00 |  | 7.14 |  | 7.14 |  | 7.14 |  |
| Antibiotherapy (days) | > 3 (n=7) | 7.14 | 0.500 | 0.00 | 0.499 | 7.14 | 0.500 | 14.29 | 0.231 |
|  | < 3 (n=7) | 0.00 |  | 7.14 |  | 0.00 |  | 0.00 |  |
| Parenteral nutrition (days) | 0 (n=3) | 0.00 | 0.571 | 7.14 | 0.214 | 0.00 | 1.000 | 0.00 | 0.999 |
|  | 0-5 (n=6) | 0.00 |  | 0.00 |  | 7.14 |  | 7.14 |  |
|  | > 5 (n=5) | 7.14 |  | 0.00 |  | 0.00 |  | 7.14 |  |
| Nasogastric feeding tube (days) | < 50 (n=9) | 0.00 | 0.571 | 7.14 | 0.643 | 0.00 | 0.571 | 0.00 | 0.110 |
|  | > 50 (n=5) | 7.14 |  | 0.00 |  | 7.14 |  | 14.29 |  |
| Mechanical ventilation | Yes (n=7) | 7.14 | 0.500 | 7.14 | 0.499 | 7.14 | 0.500 | 14.29 | 0.231 |
|  | No (n=7) | 0.00 |  | 0.00 |  | 0.00 |  | 0.00 |  |
| Hospital stay (days) | < 35 (n=5) | 0.00 | 0.643 | 7.14 | 0.357 | 0.00 | 0.643 | 0.00 | 0.396 |
|  | > 35 (n=9) | 7.14 |  | 0.00 |  | 7.14 |  | 14.29 |  |
| Meconium expulsion (h) | < 12 (n=7) | 0.00 | 0.500 | 7.14 | 0.499 | 0.00 | 0.500 | 0.00 | 0.231 |
|  | > 12 (n=7) | 7.14 |  | 0.00 |  | 7.14 |  | 14.29 |  |
| Type of feeding | Breast-fed (n=7) | 7.14 | 0.500 | 7.14 | 0.499 | 0.00 | 0.500 | 0.00 | 0.231 |
|  | Mixed-fed (n=7) | 0.00 |  | 0.00 |  | 7.14 |  | 14.28 |  |

n, number of samples.

*Percentage of samples where a genus or microbial group was isolated at least two weeks.

Significant associations are underlined (*P* values ≤ 0.05).
