# Supplementary material for: Bacterial Diversity in Meconium of Preterm Neonates and Evolution of Their Fecal Microbiota during the First Month of Life
Source: PLoS One. 2013 Jun 28;8(6):e66986. doi: 10.1371/journal.pone.0066986 (PMC3695978; doi:10.1371/journal.pone.0066986)
Supplement: Table S4 — Dominant phylotypes in meconium and 3rd week fecal samples detected by HITChip. (DOCX) [file pone.0066986.s004.docx]

Table S4. Dominant phylotypes in meconium and 3^rd^ week fecal samples.

|  | Meconium samples | | | | | | | | | | | | 3^rd^ week fecal samples | | | | | | | | | | | | | |
| --- | --- | --- | --- | --- | --- | --- | --- | --- | --- | --- | --- | --- | --- | --- | --- | --- | --- | --- | --- | --- | --- | --- | --- | --- | --- | --- |
| Species-like phylotype | n | 1 | 2 | 3 | 4 | 5 | 7 | 8 | 10 | 11 | 12 | 13 | n | 1 | 2 | 3 | 4 | 5 | 6 | 7 | 9 | 10 | 11 | 12 | 13 | 14 |
| *Enterobacter cloacae* | 5 | 0.93 | 0.57 | 1.64 | 2.29 | 5.27 | 0.03 | 0.00 | 0.00 | 0.14 | 7.60 | 1.83 | 12 | 4.83 | 1.70 | 1.14 | 1.90 | 3.37 | 4.68 | 2.90 | 1.61 | 7.19 | 2.87 | 4.90 | 0.47 | 1.38 |
| *Enterococcus faecalis* | 4 | 1.98 | 0.07 | 9.07 | 1.35 | 0.57 | 0.37 | 0.08 | 0.00 | 0.05 | 0.01 | 1.24 | 11 | 1.02 | 1.91 | 0.35 | 2.00 | 3.05 | 11.74 | 2.71 | 0.45 | 4.93 | 1.58 | 7.13 | 6.97 | 2.77 |
| *Escherichia coli* | 3 | 2.14 | 0.06 | 1.61 | 0.38 | 2.01 | 0.01 | 0.00 | 0.00 | 0.11 | 0.41 | 0.01 | 10 | 0.79 | 20.44 | 6.70 | 9.90 | 8.83 | 0.91 | 2.32 | 17.19 | 8.67 | 7.04 | 14.80 | 1.06 | 0.00 |
| *Hafnia alvei* | 5 | 0.74 | 0.08 | 6.31 | 0.54 | 10.54 | 0.07 | 0.00 | 0.00 | 1.18 | 11.02 | 2.26 | 10 | 9.23 | 0.13 | 0.70 | 1.81 | 4.13 | 12.96 | 6.43 | 3.64 | 5.50 | 12.98 | 4.80 | 0.49 | 11.17 |
| *Klebsiella pneumoniae* subsp*.ozaenae* | 4 | 0.80 | 0.57 | 2.86 | 2.28 | 5.15 | 0.04 | 0.00 | 0.00 | 0.28 | 5.63 | 0.74 | 11 | 4.41 | 1.63 | 0.33 | 2.11 | 3.63 | 3.79 | 2.25 | 1.97 | 1.91 | 4.11 | 4.18 | 0.42 | 2.75 |
| *Lactobacillus fermentum* | 8 | 9.74 | 1.96 | 2.78 | 11.91 | 1.84 | 3.13 | 0.08 | 0.02 | 24.08 | 1.40 | 0.21 | 0 | 0.00 | 0.00 | 0.00 | 0.01 | 0.01 | 0.00 | 0.00 | 0.00 | 0.00 | 0.00 | 0.00 | 0.00 | 0.00 |
| *Lactobacillus reuteri* | 3 | 0.54 | 44.89 | 0.38 | 4.00 | 0.12 | 0.21 | 0.01 | 0.00 | 1.03 | 0.10 | 0.04 | 0 | 0.00 | 0.00 | 0.00 | 0.00 | 0.00 | 0.00 | 0.00 | 0.00 | 0.00 | 0.00 | 0.00 | 0.00 | 0.00 |
| *Serratia liquefaciens* | 4 | 0.79 | 0.57 | 2.81 | 2.27 | 4.91 | 0.04 | 0.00 | 0.00 | 0.28 | 5.22 | 0.73 | 11 | 4.18 | 1.63 | 0.31 | 2.11 | 3.63 | 3.62 | 2.18 | 1.84 | 1.58 | 4.02 | 3.99 | 0.42 | 2.51 |
| *Shigella dysenteriae* | 2 | 0.61 | 0.05 | 0.68 | 0.10 | 3.52 | 0.03 | 0.00 | 0.00 | 0.05 | 4.86 | 0.10 | 11 | 3.40 | 6.64 | 1.24 | 4.16 | 4.58 | 1.40 | 1.74 | 5.45 | 2.60 | 4.30 | 6.15 | 0.50 | 0.00 |
| *Staphylococcus epidermidis* | 5 | 1.12 | 0.35 | 3.86 | 0.14 | 0.08 | 34.57 | 51.66 | 0.02 | 0.12 | 0.05 | 34.62 | 1 | 0.05 | 0.28 | 0.03 | 0.11 | 0.01 | 0.74 | 2.66 | 0.01 | 0.13 | 0.01 | 0.03 | 0.07 | 0.07 |
| *Streptococcus viridans* | 6 | 1.87 | 0.05 | 7.21 | 3.60 | 0.15 | 9.95 | 0.27 | 58.37 | 28.43 | 0.08 | 0.65 | 1 | 0.00 | 0.00 | 0.00 | 0.00 | 0.00 | 0.00 | 1.99 | 0.00 | 0.00 | 0.00 | 0.00 | 0.00 | 0.00 |
| Unc. *Streptococcus* sp. NB4D2 | 4 | 0.39 | 0.02 | 1.48 | 0.85 | 0.02 | 1.96 | 0.03 | 34.61 | 19.94 | 0.02 | 0.10 | 0 | 0.00 | 0.00 | 0.00 | 0.00 | 0.00 | 0.00 | 0.00 | 0.00 | 0.00 | 0.00 | 0.00 | 0.00 | 0.00 |
| Total contribution |  | 21.66 | 49.23 | 40.67 | 29.71 | 34.19 | 50.40 | 52.14 | 93.03 | 75.68 | 36.40 | 42.55 |  | 27.91 | 34.38 | 10.80 | 24.10 | 31.22 | 39.84 | 25.19 | 32.16 | 32.52 | 36.92 | 45.99 | 10.41 | 20.66 |

n, number of samples were the phylotype contribute for, at least, 1% of the hybridization’s signals.
