# Supplementary material for: Bacterial Diversity in Meconium of Preterm Neonates and Evolution of Their Fecal Microbiota during the First Month of Life
Source: PLoS One. 2013 Jun 28;8(6):e66986. doi: 10.1371/journal.pone.0066986 (PMC3695978; doi:10.1371/journal.pone.0066986)
Supplement: Table S5 — Proteobacterial phylotypes detected in meconium and 3rd week fecal samples using HITChip technique. (DOCX) [file pone.0066986.s005.docx]

Table S5. Proteobacterial phylotypes detected in meconium and 3^rd^ week fecal samples.

|  | Meconium samples | | | | | | | | | | | | 3^rd^ week fecal samples | | | | | | | | | | | | | |
| --- | --- | --- | --- | --- | --- | --- | --- | --- | --- | --- | --- | --- | --- | --- | --- | --- | --- | --- | --- | --- | --- | --- | --- | --- | --- | --- |
| Species-like phylotype | n | 1 | 2 | 3 | 4 | 5 | 7 | 8 | 10 | 11 | 12 | 13 | n | 1 | 2 | 3 | 4 | 5 | 6 | 7 | 9 | 10 | 11 | 12 | 13 | 14 |
| *Citrobacter amalonaticus* | 2 | 0.36 | 0.03 | 0.52 | 0.08 | 2.04 | 0.01 | 0.00 | 0.00 | 0.03 | 1.71 | 0.04 | 9 | 1.51 | 4.26 | 0.93 | 3.18 | 2.47 | 0.65 | 1.27 | 6.67 | 2.96 | 3.47 | 2.60 | 0.30 | 0.00 |
| *Citrobacter braakii* | 1 | 0.17 | 0.01 | 0.33 | 0.05 | 0.40 | 0.01 | 0.00 | 0.00 | 0.03 | 1.92 | 0.33 | 3 | 0.57 | 0.05 | 0.52 | 0.06 | 0.05 | 1.56 | 0.40 | 0.25 | 3.28 | 0.39 | 0.55 | 0.03 | 1.73 |
| *Citrobacter farmer* | 2 | 0.23 | 0.01 | 0.29 | 0.04 | 1.46 | 0.01 | 0.00 | 0.00 | 0.01 | 1.71 | 0.04 | 8 | 1.29 | 2.54 | 0.45 | 2.14 | 1.97 | 0.54 | 0.41 | 2.44 | 1.25 | 1.81 | 1.80 | 0.20 | 0.00 |
| *Citrobacter freundii* | 1 | 0.16 | 0.01 | 0.27 | 0.05 | 0.16 | 0.01 | 0.00 | 0.00 | 0.03 | 1.52 | 0.33 | 3 | 0.34 | 0.05 | 0.50 | 0.06 | 0.05 | 1.39 | 0.33 | 0.12 | 2.95 | 0.31 | 0.36 | 0.03 | 1.49 |
| *Citrobacter murliniae* | 1 | 0.17 | 0.01 | 0.33 | 0.05 | 0.40 | 0.01 | 0.00 | 0.00 | 0.03 | 1.92 | 0.33 | 3 | 0.57 | 0.05 | 0.52 | 0.06 | 0.05 | 1.56 | 0.40 | 0.25 | 3.28 | 0.39 | 0.55 | 0.03 | 1.73 |
| *Citrobacter sedlakii* | 0 | 0.26 | 0.01 | 0.35 | 0.05 | 0.60 | 0.01 | 0.00 | 0.00 | 0.03 | 0.36 | 0.01 | 8 | 0.31 | 4.29 | 1.01 | 2.79 | 1.75 | 0.24 | 0.36 | 5.16 | 2.51 | 1.97 | 2.54 | 0.19 | 0.00 |
| *Citrobacter werkmanii* | 1 | 0.13 | 0.01 | 0.29 | 0.02 | 0.30 | 0.01 | 0.00 | 0.00 | 0.02 | 1.72 | 0.33 | 3 | 0.40 | 0.08 | 0.53 | 0.07 | 0.04 | 1.24 | 0.26 | 0.26 | 4.56 | 0.32 | 0.37 | 0.03 | 1.56 |
| *Enterobacter aerogenes* | 3 | 0.17 | 0.02 | 1.60 | 0.07 | 1.73 | 0.01 | 0.00 | 0.00 | 0.17 | 1.78 | 0.06 | 6 | 1.41 | 0.02 | 0.13 | 0.40 | 0.73 | 1.76 | 0.88 | 0.75 | 1.31 | 1.43 | 1.08 | 0.06 | 1.77 |
| *Enterobacter cloacae** | 5 | 0.93 | 0.57 | 1.64 | 2.29 | 5.27 | 0.03 | 0.00 | 0.00 | 0.14 | 7.60 | 1.83 | 12 | 4.83 | 1.70 | 1.14 | 1.90 | 3.37 | 4.68 | 2.90 | 1.61 | 7.19 | 2.87 | 4.90 | 0.47 | 1.38 |
| *Escherichia coli** | 3 | 2.14 | 0.06 | 1.61 | 0.38 | 2.01 | 0.01 | 0.00 | 0.00 | 0.11 | 0.41 | 0.01 | 10 | 0.79 | 20.44 | 6.70 | 9.90 | 8.83 | 0.91 | 2.32 | 17.19 | 8.67 | 7.04 | 14.80 | 1.06 | 0.00 |
| *Escherichia fergusonii* | 0 | 0.61 | 0.02 | 0.69 | 0.11 | 0.94 | 0.01 | 0.00 | 0.00 | 0.06 | 0.41 | 0.01 | 8 | 0.37 | 8.03 | 2.10 | 3.75 | 3.24 | 0.32 | 0.70 | 6.93 | 3.32 | 2.78 | 5.29 | 0.36 | 0.00 |
| *Hafnia alvei** | 5 | 0.74 | 0.08 | 6.31 | 0.54 | 10.54 | 0.07 | 0.00 | 0.00 | 1.18 | 11.02 | 2.26 | 10 | 9.23 | 0.13 | 0.70 | 1.81 | 4.13 | 12.96 | 6.43 | 3.64 | 5.50 | 12.98 | 4.80 | 0.49 | 11.17 |
| *Klebsiella pneumoniae* | 3 | 0.68 | 0.03 | 2.11 | 0.27 | 4.25 | 0.03 | 0.00 | 0.00 | 0.20 | 4.32 | 0.71 | 10 | 3.33 | 1.06 | 0.19 | 1.61 | 2.46 | 3.06 | 1.94 | 1.68 | 0.90 | 3.51 | 3.02 | 0.30 | 1.30 |
| *K. pneumoniae* subsp*. ozaenae** | 4 | 0.80 | 0.57 | 2.86 | 2.28 | 5.15 | 0.04 | 0.00 | 0.00 | 0.28 | 5.63 | 0.74 | 11 | 4.41 | 1.63 | 0.33 | 2.11 | 3.63 | 3.79 | 2.25 | 1.97 | 1.91 | 4.11 | 4.18 | 0.42 | 2.75 |
| *Pseudomonas aeruginosa* | 1 | 0.11 | 0.54 | 0.69 | 2.03 | 0.67 | 0.01 | 0.00 | 0.00 | 0.09 | 0.90 | 0.03 | 2 | 0.85 | 0.57 | 0.12 | 0.49 | 1.16 | 0.56 | 0.25 | 0.16 | 0.68 | 0.52 | 0.97 | 0.12 | 1.20 |
| *Pseudomonas stutzeri* | 1 | 0.11 | 0.54 | 0.71 | 2.05 | 0.67 | 0.01 | 0.00 | 0.00 | 0.11 | 0.91 | 0.04 | 2 | 0.85 | 0.57 | 0.13 | 0.50 | 1.16 | 0.63 | 0.25 | 0.16 | 0.79 | 0.56 | 0.98 | 0.12 | 1.29 |
| *Salmonella enterica* subsp*. arizonae* | 2 | 0.26 | 0.00 | 0.32 | 0.05 | 1.33 | 0.00 | 0.00 | 0.00 | 0.02 | 2.49 | 0.06 | 8 | 1.15 | 4.27 | 0.71 | 2.44 | 1.72 | 0.48 | 0.51 | 4.89 | 2.31 | 2.22 | 2.83 | 0.20 | 0.00 |
| *S. enterica* subsp*. enterica* | 0 | 0.04 | 0.00 | 0.06 | 0.01 | 0.22 | 0.00 | 0.00 | 0.00 | 0.00 | 0.13 | 0.01 | 1 | 0.16 | 0.79 | 0.19 | 0.61 | 0.33 | 0.07 | 0.04 | 1.39 | 0.70 | 0.62 | 0.31 | 0.06 | 0.00 |
| *Serratia liquefaciens** | 4 | 0.79 | 0.57 | 2.81 | 2.27 | 4.91 | 0.04 | 0.00 | 0.00 | 0.28 | 5.22 | 0.73 | 11 | 4.18 | 1.63 | 0.31 | 2.11 | 3.63 | 3.62 | 2.18 | 1.84 | 1.58 | 4.02 | 3.99 | 0.42 | 2.51 |
| *Serratia marcescens* | 2 | 0.11 | 0.54 | 0.79 | 2.01 | 0.67 | 0.01 | 0.00 | 0.00 | 0.09 | 1.00 | 0.03 | 2 | 0.86 | 0.58 | 0.18 | 0.50 | 1.17 | 0.89 | 0.27 | 0.17 | 1.10 | 0.60 | 0.99 | 0.12 | 1.53 |
| *Shigella dysenteriae** | 2 | 0.61 | 0.05 | 0.68 | 0.10 | 3.52 | 0.03 | 0.00 | 0.00 | 0.05 | 4.86 | 0.10 | 11 | 3.40 | 6.64 | 1.24 | 4.16 | 4.58 | 1.40 | 1.74 | 5.45 | 2.60 | 4.30 | 6.15 | 0.50 | 0.00 |
| *Yersinia bercovieri* | 1 | 0.11 | 0.00 | 0.18 | 0.03 | 0.69 | 0.00 | 0.00 | 0.00 | 0.01 | 1.30 | 0.02 | 0 | 0.67 | 0.92 | 0.21 | 0.51 | 0.55 | 0.36 | 0.26 | 0.94 | 0.69 | 0.65 | 0.98 | 0.05 | 0.25 |
| *Yersinia frederiksenii* | 1 | 0.06 | 0.00 | 0.13 | 0.02 | 0.60 | 0.00 | 0.00 | 0.00 | 0.01 | 1.05 | 0.02 | 0 | 0.58 | 0.30 | 0.06 | 0.20 | 0.33 | 0.31 | 0.18 | 0.24 | 0.37 | 0.39 | 0.53 | 0.03 | 0.25 |
| *Yersinia rohdei* | 1 | 0.07 | 0.00 | 0.12 | 0.02 | 0.70 | 0.00 | 0.00 | 0.00 | 0.00 | 2.91 | 0.06 | 2 | 1.00 | 0.30 | 0.12 | 0.20 | 0.33 | 0.43 | 0.23 | 0.32 | 1.06 | 0.53 | 0.67 | 0.03 | 0.41 |
| Total contribution |  | 9.82 | 3.70 | 25.69 | 14.88 | 49.24 | 0.35 | 0.02 | 0.01 | 2.97 | 62.82 | 8.13 |  | 43.08 | 60.91 | 18.99 | 41.57 | 47.72 | 43.41 | 26.76 | 64.51 | 61.45 | 57.78 | 65.26 | 5.65 | 32.33 |

n, number of samples were a given phylotype contribute for, at least, 1% of the hybridization’s signals.

*Dominant phylotypes in meconium and 3^rd^ week fecal samples.
