# Supplementary material for: Bacterial Diversity in Meconium of Preterm Neonates and Evolution of Their Fecal Microbiota during the First Month of Life
Source: PLoS One. 2013 Jun 28;8(6):e66986. doi: 10.1371/journal.pone.0066986 (PMC3695978; doi:10.1371/journal.pone.0066986)
Supplement: Table S6 — Lactobacilli and lactococci phylotypes detected in meconium and 3rd week fecal samples using HITChip technique. (DOCX) [file pone.0066986.s006.docx]

Table S6. Lactobacilli and lactococci phylotypes detected in meconium and 3^rd^ week fecal samples.

|  | Meconium samples | | | | | | | | | | | | 3^rd^ week fecal samples | | | | | | | | | | | | | |
| --- | --- | --- | --- | --- | --- | --- | --- | --- | --- | --- | --- | --- | --- | --- | --- | --- | --- | --- | --- | --- | --- | --- | --- | --- | --- | --- |
| Species-like phylotype | n | 1 | 2 | 3 | 4 | 5 | 7 | 8 | 10 | 11 | 12 | 13 | n | 1 | 2 | 3 | 4 | 5 | 6 | 7 | 9 | 10 | 11 | 12 | 13 | 14 |
| *Lactobacillus antri* | 2 | 0.95 | 0.83 | 0.30 | 1.28 | 0.15 | 0.45 | 0.02 | 0.00 | 1.95 | 0.20 | 0.03 | 0 | 0.00 | 0.00 | 0.00 | 0.00 | 0.00 | 0.00 | 0.00 | 0.00 | 0.00 | 0.00 | 0.00 | 0.00 | 0.00 |
| *Lactobacillus fermentum** | 8 | 9.74 | 1.96 | 2.78 | 11.91 | 1.84 | 3.13 | 0.08 | 0.02 | 24.08 | 1.40 | 0.21 | 0 | 0.00 | 0.00 | 0.00 | 0.01 | 0.01 | 0.00 | 0.00 | 0.00 | 0.00 | 0.00 | 0.00 | 0.00 | 0.00 |
| *Lactobacillus gasseri* | 0 | 0.00 | 0.04 | 0.06 | 0.18 | 0.00 | 0.02 | 0.00 | 0.04 | 0.09 | 0.00 | 0.07 | 1 | 0.00 | 0.00 | 0.00 | 0.00 | 0.00 | 0.00 | 1.46 | 0.00 | 0.00 | 0.41 | 0.00 | 0.00 | 0.00 |
| *Lactobacillus gastricus* | 4 | 1.02 | 2.54 | 0.33 | 1.88 | 0.16 | 0.46 | 0.02 | 0.00 | 2.16 | 0.21 | 0.03 | 0 | 0.00 | 0.00 | 0.00 | 0.00 | 0.00 | 0.00 | 0.00 | 0.00 | 0.00 | 0.00 | 0.00 | 0.00 | 0.00 |
| *Lactobacillus johnsonii* | 0 | 0.00 | 0.03 | 0.05 | 0.16 | 0.00 | 0.02 | 0.00 | 0.04 | 0.09 | 0.00 | 0.06 | 1 | 0.00 | 0.00 | 0.00 | 0.00 | 0.00 | 0.00 | 1.29 | 0.00 | 0.00 | 0.35 | 0.00 | 0.00 | 0.00 |
| *Lactobacillus lactis* | 1 | 0.10 | 1.36 | 0.04 | 0.15 | 0.02 | 0.02 | 0.00 | 0.01 | 0.19 | 0.02 | 0.00 | 0 | 0.00 | 0.00 | 0.00 | 0.00 | 0.00 | 0.01 | 0.01 | 0.00 | 0.00 | 0.00 | 0.00 | 0.00 | 0.01 |
| *Lactobacillus mucosae* | 4 | 2.68 | 1.13 | 0.86 | 4.10 | 0.57 | 0.98 | 0.04 | 0.01 | 4.98 | 0.43 | 0.07 | 0 | 0.00 | 0.00 | 0.00 | 0.00 | 0.00 | 0.00 | 0.00 | 0.00 | 0.00 | 0.00 | 0.00 | 0.00 | 0.00 |
| *Lactobacillus oris* | 2 | 0.95 | 0.82 | 0.30 | 1.28 | 0.15 | 0.45 | 0.02 | 0.00 | 1.95 | 0.20 | 0.03 | 0 | 0.00 | 0.00 | 0.00 | 0.00 | 0.00 | 0.00 | 0.00 | 0.00 | 0.00 | 0.00 | 0.00 | 0.00 | 0.00 |
| *Lactobacillus reuteri** | 3 | 0.54 | 44.89 | 0.38 | 4.00 | 0.12 | 0.21 | 0.01 | 0.00 | 1.03 | 0.10 | 0.04 | 0 | 0.00 | 0.00 | 0.00 | 0.00 | 0.00 | 0.00 | 0.00 | 0.00 | 0.00 | 0.00 | 0.00 | 0.00 | 0.00 |
| *Lactobacillus salivarius* | 1 | 0.47 | 0.22 | 0.30 | 11.30 | 0.02 | 0.19 | 0.04 | 0.00 | 0.15 | 0.01 | 0.21 | 0 | 0.02 | 0.14 | 0.04 | 0.10 | 0.06 | 0.87 | 0.15 | 0.01 | 0.10 | 0.05 | 0.26 | 0.41 | 0.58 |
| *Lactobacillus salivarius* subsp. *salicinius* | 1 | 0.47 | 0.22 | 0.30 | 11.30 | 0.02 | 0.19 | 0.04 | 0.00 | 0.15 | 0.01 | 0.21 | 0 | 0.02 | 0.14 | 0.04 | 0.10 | 0.06 | 0.87 | 0.15 | 0.01 | 0.10 | 0.05 | 0.26 | 0.41 | 0.58 |
| *Lactobacillus vaginalis* | 1 | 0.04 | 12.89 | 0.06 | 0.32 | 0.01 | 0.02 | 0.00 | 0.00 | 0.16 | 0.01 | 0.00 | 0 | 0.00 | 0.00 | 0.00 | 0.00 | 0.00 | 0.00 | 0.00 | 0.00 | 0.00 | 0.00 | 0.00 | 0.00 | 0.00 |
| *Lactobacillus vaginalis* KC19 | 2 | 0.54 | 14.46 | 0.14 | 1.21 | 0.09 | 0.17 | 0.00 | 0.00 | 0.81 | 0.10 | 0.02 | 0 | 0.00 | 0.00 | 0.00 | 0.00 | 0.00 | 0.00 | 0.00 | 0.00 | 0.00 | 0.00 | 0.00 | 0.00 | 0.00 |
| *Lactococcus lactis* | 2 | 2.29 | 0.19 | 0.05 | 0.76 | 0.07 | 0.39 | 0.01 | 0.01 | 0.02 | 0.00 | 1.73 | 0 | 0.00 | 0.00 | 0.00 | 0.00 | 0.00 | 0.00 | 0.00 | 0.00 | 0.00 | 0.00 | 0.00 | 0.00 | 0.00 |
| *Lactococcus lactis* subsp*. cremoris* | 3 | 5.91 | 0.43 | 0.09 | 1.14 | 0.16 | 0.98 | 0.01 | 0.01 | 0.01 | 0.00 | 3.64 | 0 | 0.00 | 0.00 | 0.00 | 0.00 | 0.00 | 0.00 | 0.00 | 0.00 | 0.00 | 0.00 | 0.00 | 0.00 | 0.00 |
| Total contribution |  | 25.69 | 82.00 | 6.02 | 50.97 | 3.38 | 7.69 | 0.28 | 0.15 | 37.82 | 2.69 | 6.37 |  | 0.05 | 0.28 | 0.07 | 0.22 | 0.12 | 1.76 | 3.07 | 0.02 | 0.20 | 0.87 | 0.52 | 0.83 | 1.18 |

n, number of samples were a given phylotype contribute for, at least, 1% of the hybridization’s signals.

*Dominant phylotypes in meconium.
