# Supplementary material for: Bacterial Diversity in Meconium of Preterm Neonates and Evolution of Their Fecal Microbiota during the First Month of Life
Source: PLoS One. 2013 Jun 28;8(6):e66986. doi: 10.1371/journal.pone.0066986 (PMC3695978; doi:10.1371/journal.pone.0066986)
Supplement: Table S7 — Bifidobacterial phylotypes detected in meconium and 3rd week fecal samples using HITChip technique. (DOCX) [file pone.0066986.s007.docx]

Table S7. Bifidobacterial phylotypes detected in meconium and 3^rd^ week fecal samples.

|  | Meconium samples | | | | | | | | | | | | 3^rd^ week fecal samples | | | | | | | | | | | | | |
| --- | --- | --- | --- | --- | --- | --- | --- | --- | --- | --- | --- | --- | --- | --- | --- | --- | --- | --- | --- | --- | --- | --- | --- | --- | --- | --- |
| Species-like phylotype | n | 1 | 2 | 3 | 4 | 5 | 7 | 8 | 10 | 11 | 12 | 13 | n | 1 | 2 | 3 | 4 | 5 | 6 | 7 | 9 | 10 | 11 | 12 | 13 | 14 |
| *Bifidobacterium animalis* | 1 | 0.23 | 0.05 | 0.04 | 0.18 | 1.02 | 0.15 | 0.00 | 0.00 | 0.08 | 0.03 | 0.11 | 4 | 1.35 | 0.00 | 0.01 | 1.27 | 1.23 | 0.01 | 0.00 | 0.59 | 0.07 | 0.00 | 0.00 | 1.95 | 0.05 |
| *Bifidobacterium bifidum* | 1 | 0.01 | 0.01 | 0.02 | 0.07 | 1.55 | 0.02 | 0.00 | 0.00 | 0.03 | 0.00 | 0.01 | 5 | 1.43 | 0.00 | 0.01 | 1.08 | 1.13 | 0.00 | 0.00 | 1.87 | 0.04 | 0.00 | 0.00 | 2.31 | 0.00 |
| *Bifidobacterium breve* | 1 | 0.03 | 0.01 | 0.05 | 0.09 | 3.78 | 0.03 | 0.00 | 0.00 | 0.03 | 0.00 | 0.01 | 5 | 3.77 | 0.00 | 0.02 | 2.81 | 4.12 | 0.00 | 0.00 | 1.83 | 0.04 | 0.00 | 0.00 | 6.39 | 0.00 |
| *Bifidobacterium catenulatum* | 1 | 0.01 | 0.00 | 0.03 | 0.02 | 4.42 | 0.04 | 0.00 | 0.00 | 0.01 | 0.00 | 0.01 | 4 | 4.40 | 0.00 | 0.01 | 3.14 | 5.12 | 0.00 | 0.00 | 0.17 | 0.01 | 0.00 | 0.00 | 8.09 | 0.00 |
| *Bifidobacterium dentium* | 1 | 0.01 | 0.00 | 0.01 | 0.03 | 1.03 | 0.04 | 0.00 | 0.00 | 0.01 | 0.02 | 0.01 | 4 | 1.61 | 0.00 | 0.01 | 1.35 | 1.61 | 0.01 | 0.00 | 0.69 | 0.18 | 0.00 | 0.00 | 1.92 | 0.00 |
| *Bifidobacterium gallicum* | 0 | 0.23 | 0.05 | 0.03 | 0.15 | 0.81 | 0.13 | 0.00 | 0.00 | 0.08 | 0.03 | 0.11 | 5 | 1.44 | 0.00 | 0.01 | 1.07 | 1.23 | 0.01 | 0.00 | 1.33 | 0.03 | 0.00 | 0.00 | 1.99 | 0.05 |
| *Bifidobacterium infantis* | 1 | 0.03 | 0.01 | 0.05 | 0.09 | 4.27 | 0.03 | 0.00 | 0.00 | 0.03 | 0.00 | 0.01 | 5 | 4.44 | 0.00 | 0.02 | 3.25 | 4.76 | 0.00 | 0.00 | 3.41 | 0.07 | 0.01 | 0.01 | 7.50 | 0.00 |
| *Bifidobacterium longum* | 1 | 0.01 | 0.01 | 0.03 | 0.07 | 1.99 | 0.02 | 0.00 | 0.00 | 0.03 | 0.00 | 0.01 | 5 | 2.02 | 0.00 | 0.01 | 1.48 | 1.71 | 0.00 | 0.00 | 3.45 | 0.07 | 0.01 | 0.00 | 3.29 | 0.00 |
| *Bifidobacterium pseudocatenulatum* | 1 | 0.01 | 0.01 | 0.03 | 0.07 | 1.96 | 0.02 | 0.00 | 0.00 | 0.03 | 0.00 | 0.01 | 5 | 1.98 | 0.00 | 0.01 | 1.46 | 1.67 | 0.00 | 0.00 | 3.11 | 0.06 | 0.01 | 0.00 | 3.24 | 0.00 |
| *Bifidobacterium pseudolongum* | 1 | 0.01 | 0.01 | 0.02 | 0.07 | 1.55 | 0.02 | 0.00 | 0.00 | 0.03 | 0.00 | 0.01 | 5 | 1.43 | 0.00 | 0.01 | 1.08 | 1.13 | 0.00 | 0.00 | 1.87 | 0.04 | 0.00 | 0.00 | 2.31 | 0.00 |
| *Bifidobacterium thermophilum* | 1 | 0.01 | 0.00 | 0.01 | 0.01 | 2.02 | 0.03 | 0.00 | 0.00 | 0.01 | 0.00 | 0.01 | 4 | 1.97 | 0.00 | 0.01 | 1.32 | 2.11 | 0.00 | 0.00 | 0.07 | 0.01 | 0.00 | 0.00 | 3.24 | 0.00 |
| Unc. *Bifidobacterium* sp. 15D | 0 | 0.01 | 0.00 | 0.01 | 0.03 | 0.94 | 0.02 | 0.00 | 0.00 | 0.01 | 0.00 | 0.01 | 4 | 1.64 | 0.00 | 0.01 | 1.38 | 1.47 | 0.00 | 0.00 | 0.65 | 0.05 | 0.00 | 0.00 | 2.23 | 0.00 |
| Total contribution |  | 0.61 | 0.13 | 0.34 | 0.87 | 25.34 | 0.56 | 0.01 | 0.01 | 0.38 | 0.08 | 0.30 |  | 27.46 | 0.02 | 0.11 | 20.68 | 27.28 | 0.05 | 0.02 | 19.04 | 0.68 | 0.05 | 0.04 | 44.44 | 0.11 |

n, number of samples were a given phylotype contribute for, at least, 1% of the hybridization’s signals.
